# Clinical Value and Potential Mechanisms of Oxysterol-Binding Protein Like 3 (OSBPL3) in Human Tumors

**DOI:** 10.3389/fmolb.2021.739978

**Published:** 2021-10-19

**Authors:** Na Hao, Yudong Zhou, Yijun Li, Huimin Zhang, Bin Wang, Xiaona Liu, Yu Ren, Jianjun He, Can Zhou, Xiaojiang Tang

**Affiliations:** Department of Breast Surgery, First Affiliated Hospital of Xi’an Jiaotong University, Xi’an, China

**Keywords:** OSBPL3, cancer, prognosis, phosphorylation, immune infiltration

## Abstract

Cancer remains one of the top culprits causing disease-related deaths. A lack of effective multi-cancer therapeutic targets has limited the prolongation of cancer patients’ survival. Therefore, it is important to explore novel oncogenic genes or versatile targets and perform a comprehensive analysis to assess their roles in the process of tumorigenesis. OSBPL3 protein is an intracellular lipid receptor of the oxysterol-binding protein superfamily, which participates in some pathological and physiological processes in tumor progression. However, its clinical roles and potential mechanisms in cancers remain unknown. Thus, we aimed to systematic explore the potential oncogenic roles of OSBPL3 across thirty-three tumors using multiple web-based and publicly available tools, including the Cancer Genome Atlas, Gene Expression Omnibus, Genotype-Tissue Expression, cBioPortal, and Human Protein Atlas database. OSBPL3 is highly expressed in major subtypes of cancers, distinctly associated with the prognosis of tumor patients. We observed X676_splice/V676G alteration in the oxysterol domain and frequent mutations of OSBPL3 involve cell survival in skin cutaneous melanoma. We also first presented that the expression of OSBPL3 was associated with tumor mutational burden (TMB) in nine cancer types. Additionally, OSBPL3 shows an enhanced phosphorylation level at S426, S251, and S273 loci within the pleckstrin homology domain in multiple tumors, such as breast cancer or lung adenocarcinoma. And OSBPL3 expression was associated with active immune cells (CD8^+^ T cells) and cancer-associated fibroblasts in breast cancer, colon adenocarcinoma, and kidney renal clear cell carcinoma and immune checkpoint genes in more than 30 tumors, but weakly associated with immune suppressive cells (myeloid-derived suppressor cells, T regulatory cells). Moreover, protein processing and mRNA metabolic signaling pathways were involved in the functional mechanisms of OSBPL3. Our study first demonstrated that a novel agent OSBPL3 plays an important role in tumorigenesis from the perspective of publicly available databases and clinical tumor samples in various cancers, which comprehensively provide insights into its biological functions and may be helpful for further investigation.

## Introduction

Given the complexity of carcinogenesis, it is important to explore novel oncogenic genes or targets and perform a pan-cancer analysis to assess their roles in the process of tumorigenesis. In recent years, cancer genomes and databases have yielded valuable insights into the etiology of molecular processes and have fueled the promises of pan-cancer analysis, which provides a fully comprehensive understanding of genes with clinical outcomes and potential molecular mechanisms across different types of tumors (Zhang et al., 2019).

OSBPL3 (oxysterol-binding protein like 3) protein encodes a member of the oxysterol-binding protein family, a group of intracellular lipid receptors (Gregorio-King et al., 2001; Lehto et al., 2001). The “full-length” OSBPL3 gene comprises 23 exons and encodes a predicted protein of 887 amino acids with a C-terminal OSBP domain and an N-terminal pleckstrin homology (PH) domain (Collier et al., 2003). Under physiological conditions, OSBPL3 locates in the endoplasmic reticulum and plasma membrane, regulating cell adhesion, the actin cytoskeleton, vesicle transport, and cellular lipid metabolism (Lehto and Olkkonen, 2003; Lehto et al., 2005; Yan et al., 2007; Lehto et al., 2008; D’Souza et al., 2020; Gulyas et al., 2020). So far, there are few studies on OSBPL3 in tumors. Recent transcriptome analyses from the perspective of pathology and clinical pathways have suggested that OSBPL3 is expressed and involved in the development of cancers, including colorectal cancer (Xu et al., 2020; Chen et al., 2021), pancreatic ductal adenocarcinoma (Li et al., 2017), recurrent glioblastoma (Erdem-Eraslan et al., 2016), and metastatic breast cancers (Lefebvre et al., 2016). Furthermore, OSBPL3 is generally considered to be an oncogene factor in colorectal cancer progression, acting through upregulation by HIF1A and activation of the RAS signaling pathway (Jiao et al., 2020). However, there has been little clear evidence about the expression and role of OSBPL3 in tumors, and still, there is no pan-cancer study on the relationship between OSBPL3 and various cancers.

Here, we aim to conduct a comprehensive analysis of OSBPL3 based on the TCGA project and summarize a group of roles of OSBPL3 in pathogenesis, clinical outcomes, and molecular mechanisms with various cancers, including gene expression, survival prognosis, genetic alteration, protein phosphorylation, immune infiltration, and relevant cellular pathways, which provide a better understanding of OSBPL3 in tumorigenesis and will be valuable for further in-depth research.

## Materials and Methods

### Cell Culture and Human Sample Collection

MDA-MB-231 cells were cultured in L15 medium. T-47D, MCF-10A, HeLa, BT549, A549, H446, H460, SW480, HCT116, HepG2, ZR-75-1, SK-BR-3, MDA-MB-488, and MCF-7 cells were cultured in DMEM. All culture media were supplemented with 10% FBS (Hyclone). All cells except MDA-MB-231 were grown at 37°C in 5% CO_2_ incubators, and MDA-MB-231 cells were grown at 37°C in 0% CO_2_ incubators. All cells were passaged for less than 3 months before renewal from frozen, early-passage stocks. All cells were purchased from the ATCC, and all were tested to ensure that they were mycoplasma negative.

### qRT-PCR

Total RNAs were purified with the RNeasy Mini Kit (Qiagen), and cDNA was synthesized with the SuperScript III First-Strand Synthesis SuperMix for qRT-PCR (Thermo Fisher Scientific). The expression levels of OSBPL3 and GAPDH mRNA were quantified with the LightCycler 480 Real-Time PCR System with Universal ProbeLibrary Probe #36 (Roche). The primers were as follows: OSBPL3, 5′-TTG​GTG​TGT​CCC​AAA​AAT​TGG​T-3′ (forward) and 5′-TCC​TGG​GTG​TAA​TTC​ATC​TCC​C-3′ (reverse), and GAPDH, 5′-TCA​TCC​CTG​CCT​CTA​CTG-3′ (forward) and 5′-TGC​TTC​ACC​ACC​TTC​TTG-3′ (reverse).

### Western Blotting

Western blotting (WB) was performed as described previously (Hao et al., 2020) with the following modifications. The primary antibodies were OSBPL3 (sc-514097, Santa Cruz, United States) at a 1:500 dilution and β-actin (sc-47778, Santa Cruz Biotechnology) at a 1:1,200 dilution. Images were acquired using the Bio-Rad ChemiDoc MP Imaging System (Bio-Rad). All western blots were a representative image of three separate experiments.

### Immunohistochemistry and Immunofluorescence

IHC and IF staining was performed as described previously (Hao et al., 2020) with the following modifications. Antigen retrieval for sections of tissue microarrays (TMA PR803b, US Biomax, Inc.) was performed in a pressure cooker. The antibodies was both anti-OSBPL3 (sc-514097, Santa Cruz, United States).

### Expression of OSBPL3 in Various Cancers

We searched OSBPL3 on the TIMER2 web (tumor immune estimation resource, version 2) (http://timer.cistrome.org/) with the “Gene_DE” module and obtained the expression pattern of OSBPL3 between tumor and corresponding normal tissues from the TCGA project. For tumors’ lack of normal tissues [e.g., GBM (glioblastoma multiforme), LAML (acute myeloid leukemia)], we searched the GEPIA2 web server (Gene Expression Profiling Interactive Analysis, version 2) (http://gepia2.cancerpku.cn/#analysis) with the “Expression analysis-Box Plots” module from the GTEx (Genotype-Tissue Expression) database and added the expression of OSBPL3 with box plots. The related parameters are as follows: *p*-value cutoff = 0.01, log_2_FC (fold change) cutoff = 1, and “matched TCGA normal and GTEx data.” We also obtained the expression difference of OSBPL3 in pathological stages (stages I–IV) of different tumors with violin plots via the “Pathological Stage Plot” module of GEPIA2. Then, log_2_ [TPM (transcripts per million) + 1] transformation was applied.

We searched the Oncomine database (https://www.oncomine.org/resource/main.html) and obtained the expression difference data of the OSBPL3 gene between tumor and normal tissues by setting the threshold of *p*-value = 0.001, gene rank = 10%, and fold change = 2.

### Survival Prognosis Value of OSBPL3

We obtained the overall and disease-free survival data of OSBPL3 across all types of tumors via the “Survival Map” module of GEPIA2. High (50%) and low (50%) cutoff values were split into high and low expression cohorts. The log-rank test was used in the hypothesis test, and the survival plots were also plotted. We also used the interactive operation interface of the Kaplan–Meier plotter (http://kmplot.com/analysis/) to pool the GEO datasets for a series of meta-analyses of progression-free interval (PFI) and disease-free interval (DFI) survival. The hazard ratios (HRs), 95% confidence intervals (95% CIs), and log-rank *p*-values were computed, and the Kaplan–Meier survival plots were generated. The meta-analysis was statistically mapped by STATA 12.0 software (StataCorp LP, College Station, TX, United States).

### Genetic Alteration and Mutation Landscapes of OSBPL3

We queried the genetic alteration characteristics of OSBPL3 and obtained the alteration frequency, mutation site, and type and CNA (copy number alteration) across all TCGA tumors on the cBioPortal web (https://www.cbioportal.org/) with the “TCGA Pan Cancer Atlas Studies” module. The mutation sites of OSBPL3 were plotted with a schematic diagram of the protein and the 3D (three-dimensional) structure with the “Mutations” module. We also generated the data on the overall, disease-free, progression-free, and disease-free survival curves for the different cancer types with or without OSBPL3 genetic alteration by Kaplan–Meier plots via the log-rank *p*-value.

We investigated the correlation between OSBPL3 expression and tumor mutational burden (TMB)/microsatellite instability (MSI) in different tumors from the TCGA project on the web of “http://sangerbox.com/Tool.” Spearman’s rank correlation test was performed, and the *p*-value and partial correlation (cor) value are shown.

### Protein Phosphorylation Analysis

We searched the expression level of the total protein and phosphoprotein of OSBPL3 (NP_055205.2) using the CPTAC dataset. The available datasets of six tumors are shown—BRCA (breast cancer), OV (ovarian cancer), colon cancer, RCC (clear cell), UCEC (uterine corpus endometrial carcinoma), and lung adenocarcinoma.

### Immune Infiltration Analysis

We explored the association between OSBPL3 expression and immune infiltrates on the TIMER2 database with the “Immune-Gene” module and analyzed the immune score and stromal score using R software “Estimations” with Spearman’s analysis. The immune cells of neutrophils, macrophages, dendritic cells, CD8^+^ T cells, CD4^+^ T cells, B cells, myeloid-derived suppressor cells (MDSCs), T regulatory cells (Tregs), and cancer-associated fibroblasts (CAFs) were selected. The algorithms of immune infiltration estimations include TIMER, CIBERSORT, CIBERSORT-ABS, QUANTISEQ, XCELL, MCPCOUNTER, and EPIC. The results were visualized with a heatmap and a scatter plot.

### Identification of Differentially Expressed Genes and OSBPL3 Co-Expressed Genes

We obtained the top 50 available experimentally determined OSBPL3-binding proteins via searching the STRING website (https://string-db.org/). The main parameters are organism (“*Homo sapiens*”), minimum required interaction score [“low confidence (0.150)”], meaning of network edges (“evidence”), max. number of interactors to show (“no more than 50 interactors” in the first shell), and active interaction sources (“experiments”). We also obtained the top 100 OSBPL3-correlated targeting genes from GEPIA2 with the “Similar Gene Detection” module and performed a pairwise Pearson correlation analysis of OSBPL3 and selected genes applying the “correlation analysis” module of GEPIA2. The value of TPM was converted with Log_2_ for the dot plot, and the *p*-value and the correlation coefficient (R) were labeled. The heatmap of the selected genes used the “Gene_Corr” module of TIMER2, and we conducted an intersection Venn analysis to access the OSBPL3-binding genes using FUNRICH software.

### OSBPL3-Related Gene Enrichment and Molecular Mechanism Analysis

Moreover, we performed KEGG (Kyoto Encyclopedia of Genes and Genomes) and GO (Gene Ontology) enrichment analyses. Briefly, we uploaded the gene lists (above-mentioned) to DAVID (the Database for Annotation, Visualization, and Integrated Discovery) with the settings of selected identifier (“OFFICIAL_GENE_SYMBOL”) and species (“*Homo sapiens*”) and obtained the functional annotation chart. The enriched pathways were finally visualized with the “tidyr,” “ggplot2,” and “clusterProfiler” R packages. The analysis of GO enrichment including biological process (BP), cellular component (CC), and molecular function (MF) was visualized as cnetplots, using the cnetplot function (circular = F, colorEdge = T, node_label = T).

### Statistical Analysis

All the data of gene expression were normalized by log_2_ transformation. The comparison of tumor and normal tissues used two sets of *t*-tests; *p* < 0.05 indicates statistical significance. The Kaplan–Meier curve and log-rank test were used for all survival analyses. The correlation analysis between the two variables used Spearman’s or Pearson’s test. All statistical analyses were processed by R software (version 4.0.2).

## Results

### Expression Pattern of OSBPL3 in Various Cancers

In this study, we aimed to explore the roles of the human OSBPL3 gene in different cancers. We first explored the gene and protein information of OSBPL3. As shown in Supplementary Figure S1A, the OSBPL3 gene is located on chr7: 24,827,146-24,949,571 and contains four isoforms, and the OSBPL3 protein structure is relatively conserved among different species (e.g., *H. sapiens*, *P. troglodytes*, *M. mulatta*) and generally consists of the oxysterol-binding protein (Oxysterol_BP) (pfam01237) domain and pleckstrin homology (PH) domain (cl17171) (Supplementary Figure S1B). The phylogenetic tree shows the evolutionary relationship of the OSBPL3 protein between different species (Supplementary Figure S1C).

To explore the expression pattern of OSBPL3 under physiological conditions, we detected OSBPL3 expression in all types of normal tissues based on the HPA (Human Protein Atlas), GTEx, and FANTOM5 (Functional ANnoTation Of the Mammalian genome 5) datasets (Supplementary Figure S2A). OSBPL3 was expressed in all detected tissues (all consensus normalized expression values > 1) and highly expressed in the parathyroid gland followed by the appendix (Supplementary Figure S2B) but presented low RNA tissue specificity as well as low RNA cell type specificity in different normal cells (Supplementary Figure S2C).

**FIGURE 1:**
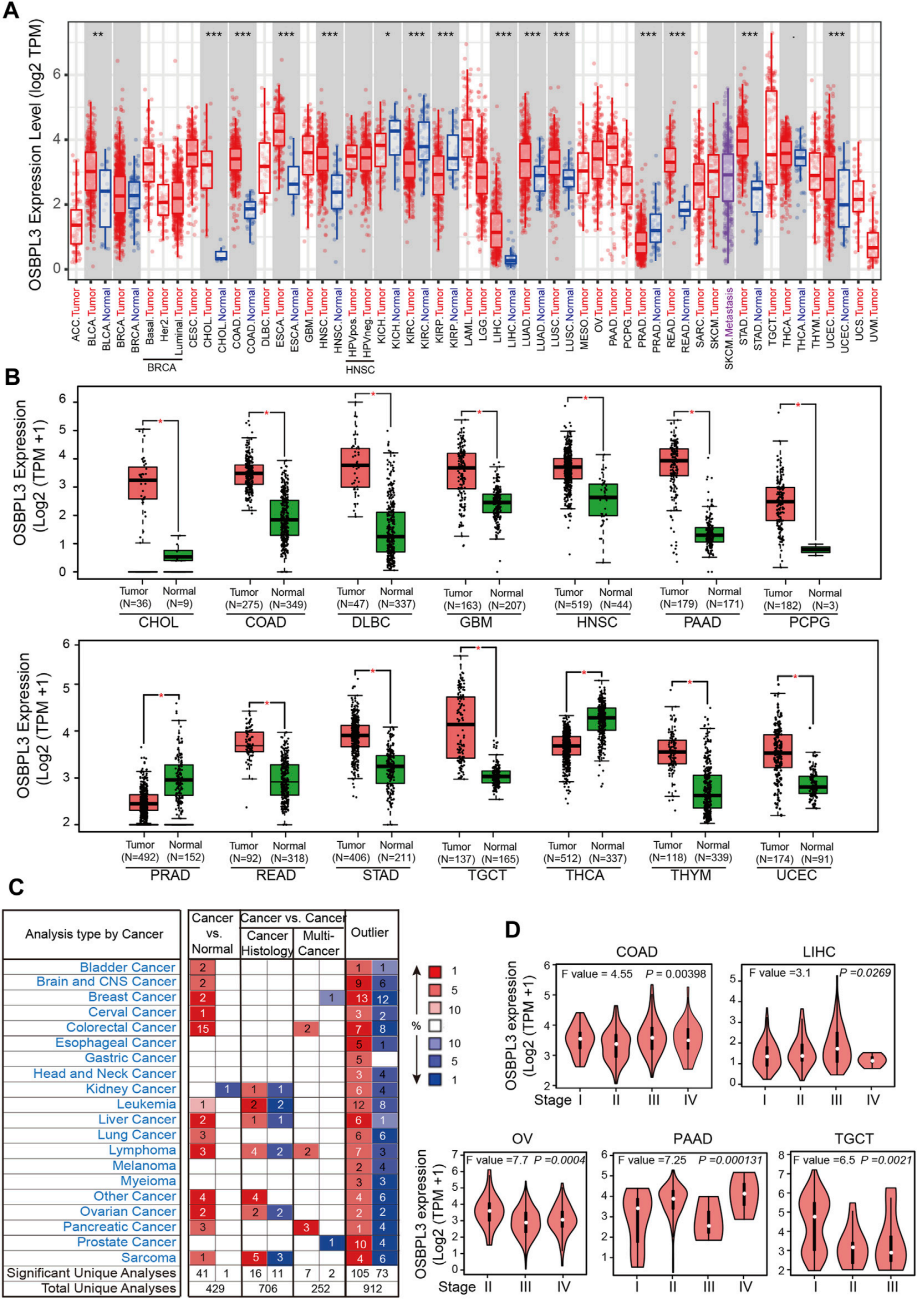
Expression level of the OSBPL3 gene in different tumors and pathological stages. **(A)** Expression status of the OSBPL3 gene across diverse cancers from the TCGA dataset in TIMER. **(B)** Expression of OSBPL3 in the cancers from the TCGA project and the corresponding normal tissues from the GTEx database of CHOL, COAD, DLBC, GBM, HNSC, PAAD, PCPG, PRAD, READ, STAD, TGCT, THCA, THYM, and UCEC. The box plot data were supplied. **(C)** Transcription levels of OSBPL3 in different types of human cancers from Oncomine. The cell number represents the dataset number that meets all of the thresholds (blue: downregulated; red: upregulated). The best gene rank percentile was applied for the analyses within the cell. **(D)** The expression levels of the OSBPL3 gene were analyzed by pathological stages (stages I–IV) of COAD, LIHC, OV, PAAD, and TGCT in the TCGA project. Log_2_ (TPM+1) transformation was applied. *p < 0.05; **p < 0.01; ***p < 0.001.

Next, we evaluated the expression status of OSBPL3 across various cancers from the TCGA project. As shown in Figure 1A, the expression level of OSBPL3 is significantly higher than the corresponding normal tissues in multiple tumor tissues except breast cancer. We also compared the expression of OSBPL3 between the tumor tissues from TCGA and the normal tissues from the GTEx dataset. Figure 1B and Supplementary Figure S3A indicate that the expression of OSBPL3 is significantly upregulated in all types of cancers except only kidney renal clear cell carcinoma (KICH). The results of the CPTAC dataset also showed similar results in cancer types of renal, colon, and lung cancers and uterine corpus endometrial carcinoma (Supplementary Figure S3B, *p* < 0.001).

Furthermore, we identified the mRNA expression of OSBPL3 across different types of human cancers and the corresponding normal tissues from the Oncomine database. Figure 1C shows that OSBPL3 expression was higher in multiple cancer groups, including bladder, brain, breast, cervical, colorectal, liver, lung, ovarian, and pancreatic cancers as well as leukemia and lymphoma. The pooling analysis results of over 40 reports confirmed that OSBPL3 is highly upregulated in different kinds of cancers than in normal tissues—pancreatic cancer, lung cancer, colorectal cancer, liver cancer, and cervical cancer (all *p* < 0.05) (Supplementary Figures S4A–E).

**FIGURE 2:**
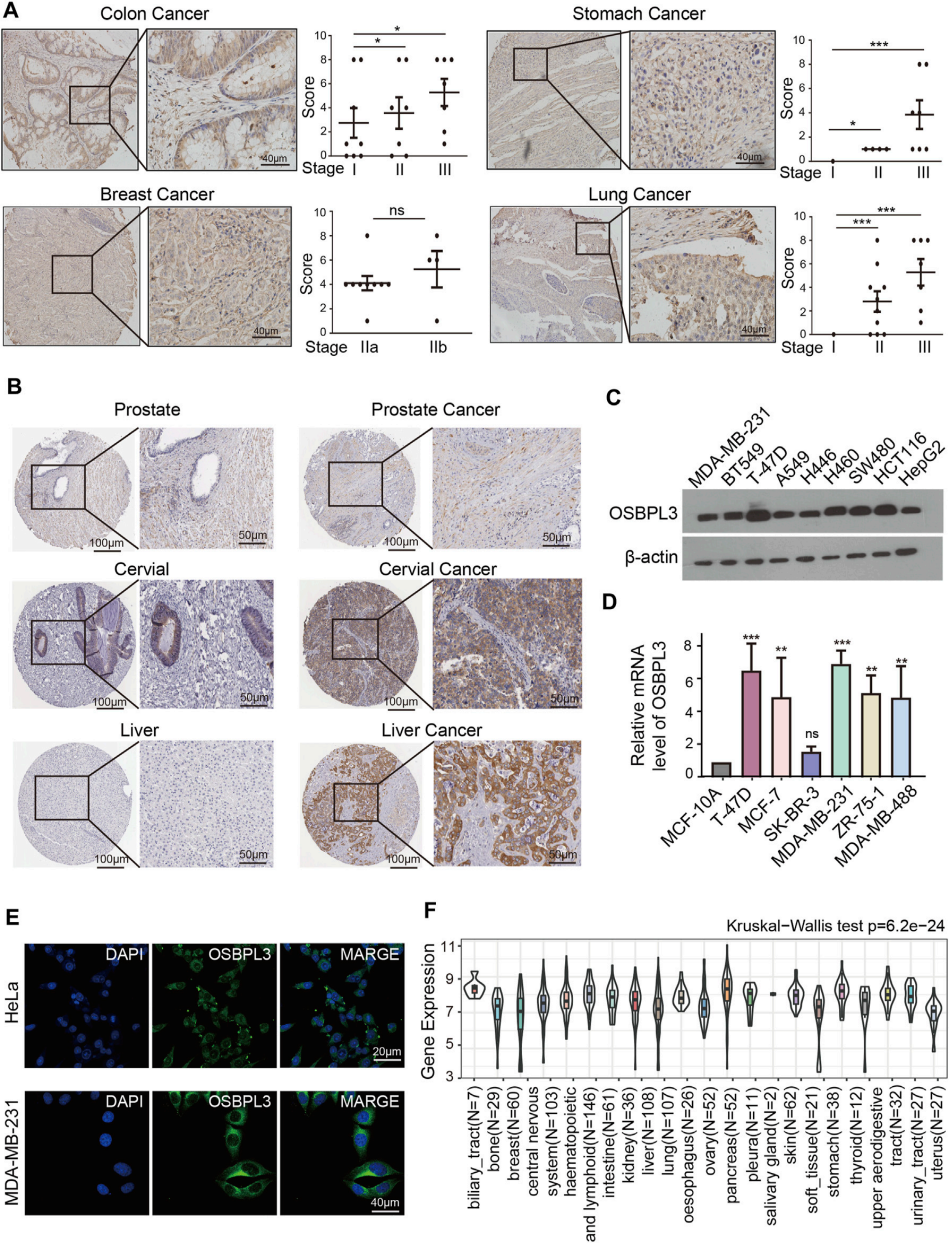
Expression and location of OSBPL3 in a variety of cancer tissues and cells. **(A)** OSBPL3 expression of multi-tissue arrays of colon cancer, stomach cancer, breast cancer, and lung cancer by IHC staining and statistical analysis of the relationship of OSBPL3 expression with tissue status. **(B)** OSBPL3 expression in different tumor tissues of prostate cancer, cervical cancer, liver cancer, and lung cancer by IHC staining from the HPA (Human Protein Atlas) database. **(C)** IF assays for subcellular localization of OSBPL3 in cervical cancer HeLa (up) and breast cancer MDA-MB-231 (down) cell lines (blue: nucleus; green: OSBPL3). **(D)** The protein expression levels of OSBPL3 in nine cancer cell lines (MDA-MB-231, BT549, T47D, A549, H446, H460, SW480, HCT116, and HepG2) were detected by WB. **(E)** The mRNA expression levels of OSBPL3 in six breast cancer cell lines (T-47D, MCF-7, SK-BR-3, MDA-MB-231, MDA-MB-468, and ZR-75-1) and one normal breast ductal epithelial cell line (MCF-10A) were detected by qRT-PCR (down). **(F)** Gene expression in various tumor cells from the CCLE database. *p < 0.05; **p < 0.01; ***p < 0.001.

To fully appreciate the expression and correlation of OSBPL3 with different cancers, we experimented the expression levels of OSBPL3 in multiple types of cancer tissues (breast, lung, stomach, and colon) using microarrays consisting of 63 biopsies (Figure 2A) and three cancer tissues (prostate, cervical, and liver) from the HPA dataset (Figure 2B) by IHC staining. The results showed that OSBPL3 was expressed at abnormally high levels in these tumor biopsies. The clinical pathological grade of a tumor cancer closely correlates with malignancy and differentiation, and we found a positive correlation between elevated expression levels of OSBPL3 and high-grade tumors in both the cancer biopsies (breast, lung, stomach, and colon cancers) (Figure 2A) and the tumor statistics data from the GEPIA2 database (COAD, LIHC, OV, PAAD, and TGCT) (Figure 1D). We also checked the protein (Figure 2C) and mRNA expression levels of OSBPL3 (Figure 2D) in four cancer cells (breast, lung, colon, and liver) and the protein expression in all types of human cancer cells from the CCLE dataset (Figure 2F), and the results showed that OSBPL3 had high expression levels in cancer cell lines. Additionally, the IF assays localized endogenous OSBPL3 to the cytoplasm (Figure 2E).

Based on the above-mentioned findings, OSBPL3 is generally upregulated in multiple human cancer tissues, in particular in digestive system carcinoma (e.g., gastrointestinal cancer, colorectal cancer, liver cancer, and pancreatic cancer) and female cancers (e.g., breast cancer, cervical cancer, and ovarian cancer), which may act as an oncogene in most cancer types.

### Survival and Prognostic Value of OSBPL3

**FIGURE 3:**
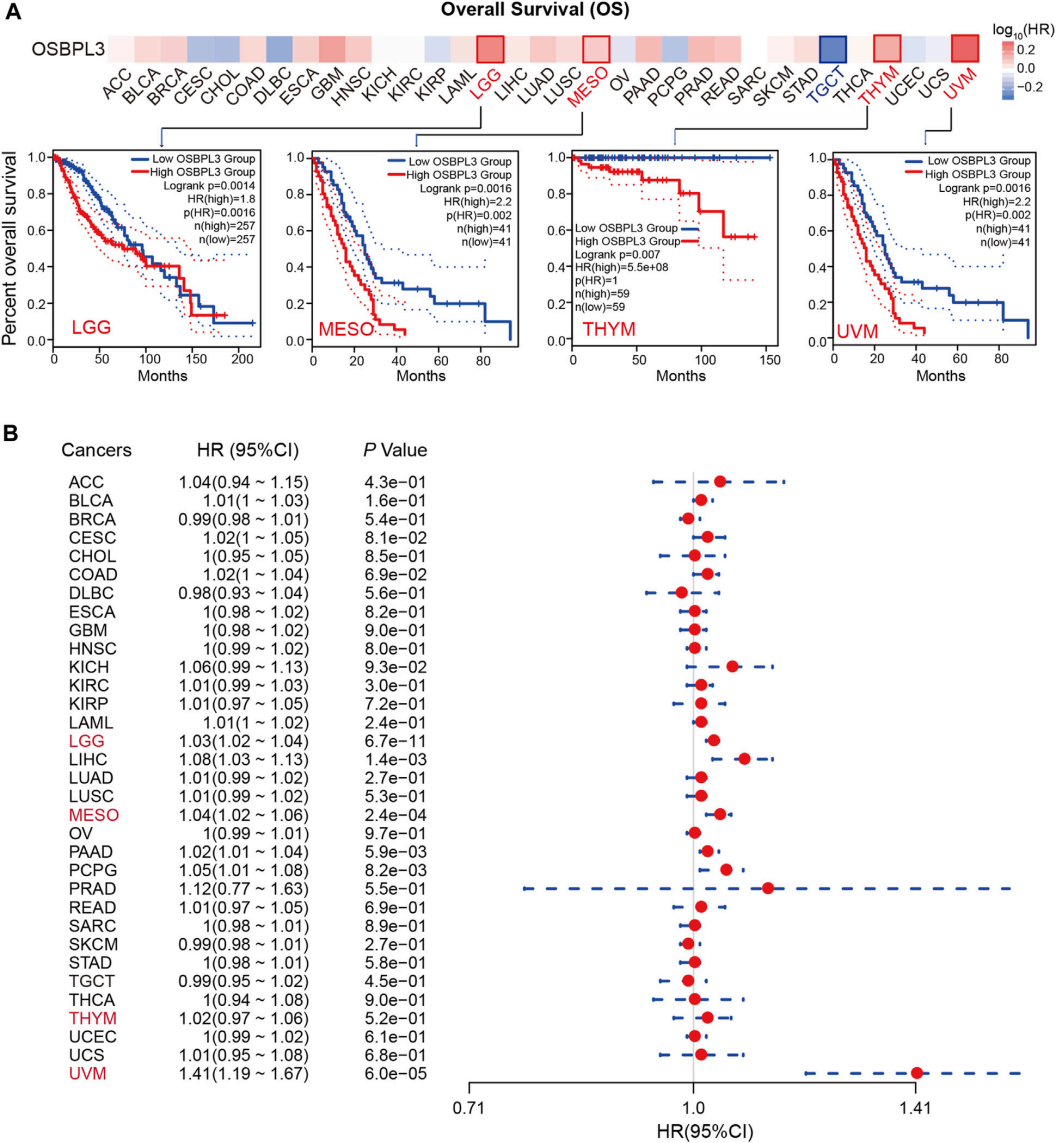
Correlation between OSBPL3 expression and overall survival prognosis of different cancers. **(A)** Overall survival (OS) analyses of OSBPL3 gene expression across different tumors in TCGA via the GEPIA2 dataset. Up: survival map; down: Kaplan–Meier plotter curves. **(B)** A meta-analysis (forest plot) for pooling of a series of univariate overall survival of OSBPL3 expression in different tumors. Hazard ratios (HRs), 95% confidence intervals (95% CIs), and *p*-values are shown.

**FIGURE 4:**
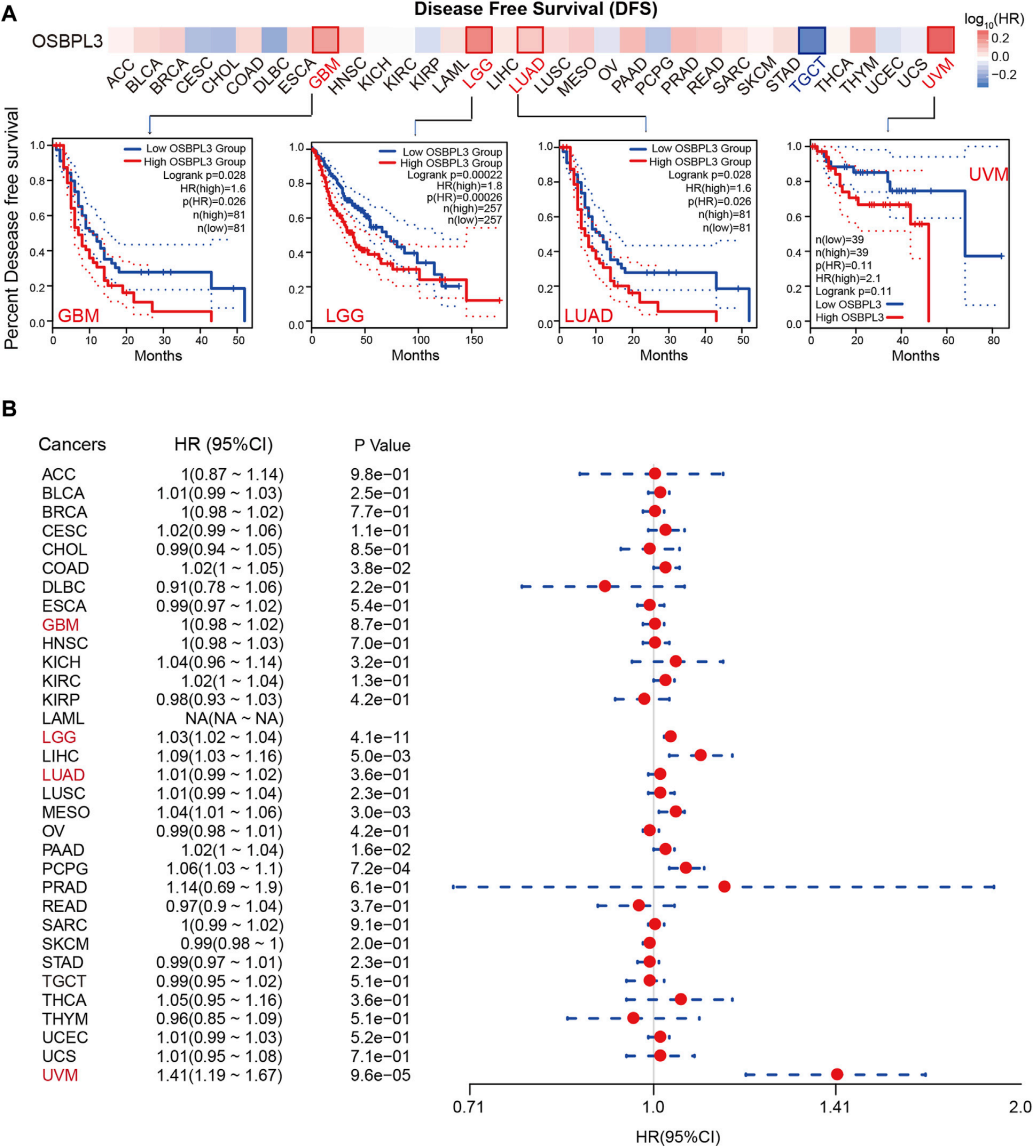
Correlation between OSBPL3 expression and disease-free survival (DFS) prognosis of different cancers. **(A**) Disease-free survival analyses of OSBPL3 gene expression across different tumors in TCGA via the GEPIA2 dataset. Up: survival map; down: Kaplan–Meier plotter curves. **(B)** A meta-analysis (forest plot) for pooling of a series of univariate disease-free survival of OSBPL3 expression in different tumors. Hazard ratios (HRs), 95% confidence intervals (95% CIs), and *p*-values are shown.

**FIGURE 5:**
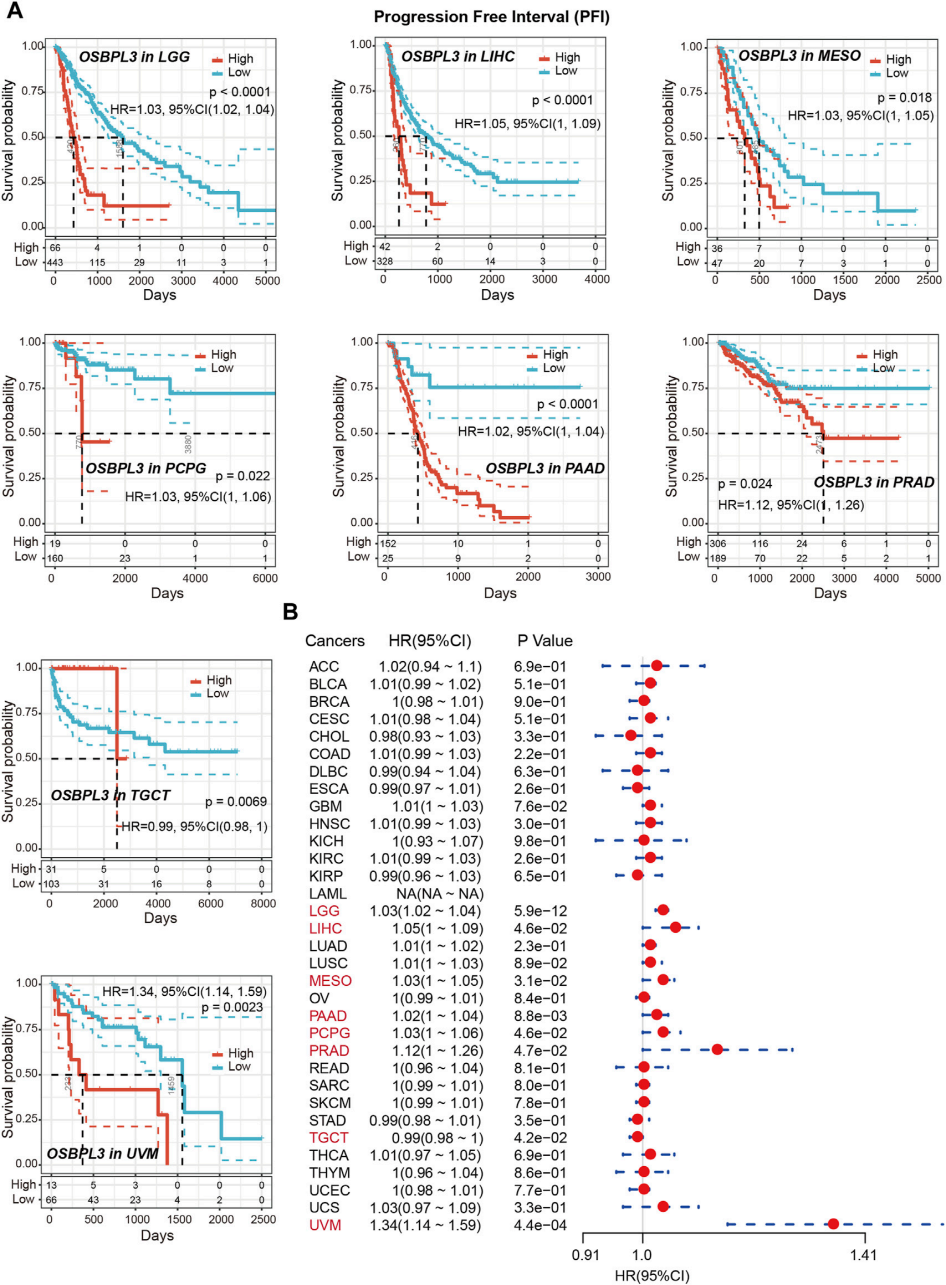
Correlation between OSBPL3 expression and progression-free survival (PFS) prognosis of cancers in TCGA. **(A**) Progression-free survival analyses of OSBPL3 gene expression by Kaplan–Meier curves in LGG, LIHC, MESO, PCPG, PAAD, PRAD, TGCT, and UVM cancers. **(B)** A meta-analysis (forest plot) for pooling of a series of univariate progression-free survival analyses of OSBPL3 expression in different tumors. Hazard ratios (HRs), 95% confidence intervals (95% CIs), and *p*-values are shown.

To determine whether the expression of OSBPL3 is associated with the outcome of patients with different tumors, we performed survival analysis using progression or deaths cases as endpoints. As shown in Figure 3A, high expression level of OSBPL3 correlates with poor prognosis of OS (overall survival) for LGG (low-grade glioma), MESO, THYM, and UVM (uveal melanoma) cancers and DFS (disease-free survival) for GBM, LGG, LUAD, and UVM cancers (all *p* < 0.05) (Figure 4A). To further evaluate the relationship between OSBPL3 and tumor progression (relapse/metastasis), we confirmed the PFI (Figure 5A) and DFI (Supplementary Figure S5A), and the data showed highly expressed OSBPL3 was linked to poor PFI and DFI for both LGG and PAAD cancers and poor PFI for LIHC, MESO, PCPG, PRAD, TGCT, and UVM cancers (all *p* < 0.05).

We also performed meta-analysis of the correlation between OSBPL3 expression and prognosis with different cancers using the Kaplan–Meier plotter (Figures 3B, 4B, 5B, Supplementary Figure S5B). The results presented a significant correlation between highly expressed OSBPL3 and poor OS, DFS, PFI, and DFI prognosis for LGG; poor OS, DFS, and PFI for UVM; and poor PFI and DFI for PAAD. In contrast, a low OSBPL3 expression level was associated with poor OS (*P* = 0.004) and DFS (*P* = 0.028) prognosis for TGCT (Figures 3A, 4A).

The above data indicated that OSBPL3 expression is significantly associated with the poor prognosis of patients with LGG, UVM, PAAD, and MESO cancers but differentially associated with other tumors and may act as a detrimental prognostic factor in these tumors, which is worth further exploration.

### Genetic Alteration and Mutation Landscapes of OSBPL3

**FIGURE 6:**
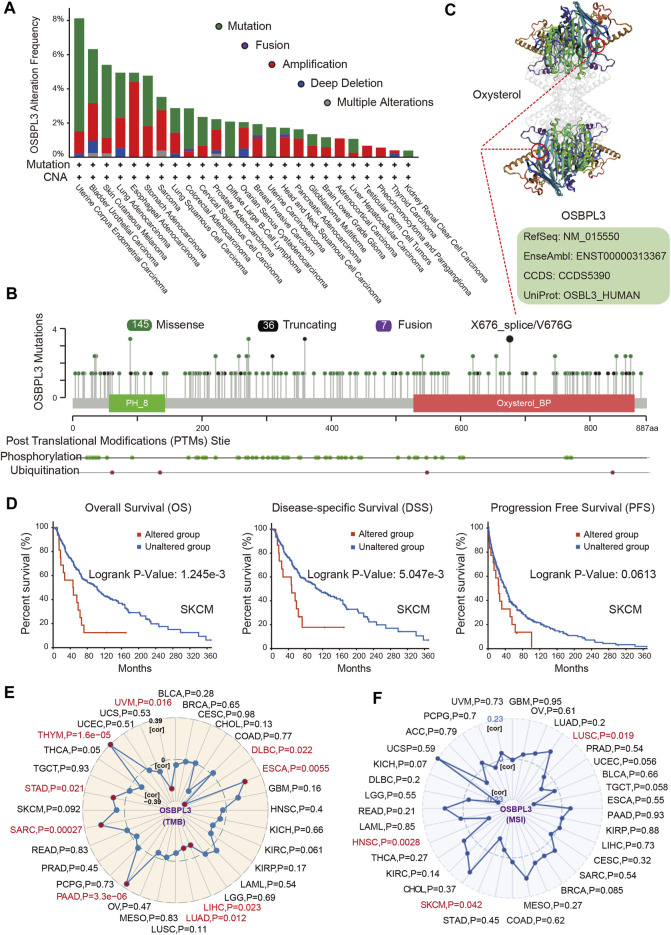
Alteration feature of OSBPL3 and correlation between OSBPL3 expression and tumor mutational burden (TMB)/microsatellite instability (MSI) in different tumors of TCGA on the cBioPortal database. **(A)** Alteration frequency with the mutation type of OSBPL3. **(B)** Alteration with the mutation site of OSBPL3. **(C)** 3D structure of the mutation site of OSBPL3 with the highest alteration frequency (X676_splice/V676G). **(D)** Correlation between the mutation status of OSBPL3 and overall, disease-specific, and progression-free survival of SCM cancer. **(E–F)** Correlation between OSBPL3 expression and TMB **(E)**/MSI **(F)** with different tumors of TCGA. The *p*-value is supplied.

Next, we investigated the genetic alteration status of OSBPL3 in various tumors of the TCGA cohorts. As shown in Figure 6A, the highest mutation frequency of OSBPL3 (>6%) appears for patients with uterine corpus endometrial carcinoma and the amplification (an alteration frequency of ∼5%) appears for the esophageal adenocarcinoma. Figure 6B shows the mutation types, sites, and case numbers of OSBPL3 and the main type of genetic alteration was the missense mutation. And the X676_splice/V676G alteration in the oxysterol domain induces a frame shift mutation of OSBPL3 with the translation from P (Proline) to L (Leucine) at the 673 site of OSBPL3 protein, and Figure 6C shows subsequent OSBPL3 protein truncation with the 3D structure. We also explored the potential association between the genetic alteration of OSBPL3 and the clinical survival outcome with different types of cancers. Figure 6D indicates that patients with altered OSBPL3 showed poorer prognosis compared with non-alteration in overall survival (*P* = 1.245e-3) and disease-specific survival (*P*= 5.047e-3), but not progression-free survival (*P* = 0.0613) in SKCM (skin cutaneous melanoma).

We also analyzed the correlation between OSBPL3 expression and tumor mutational burden (TMB)/microsatellite instability (MSI) across diverse tumors from TCGA. Figures 6E,F show a positive correlation between high OSBPL3 expression and TMB for DLBC, ESCA, LIHC, LUAD, PRAD, SARC, STAD, THYM, and UVM (all *P* = 0.05) but a positive correlation with MSI only for LUSC (*P* = 0.019), SKCM (*P* = 0.042), and HNSC (*P* = 0.0028). The meta-analysis showed the details (Supplementary Figures S6A,B). This result suggested that most of the cancers with high expression of OSBPL3 have more tumor mutation burden and less microsatellite instability, which need more sample tests for confirmation and in-depth research.

### Protein Phosphorylation Analysis of OSBPL3

**FIGURE 7:**
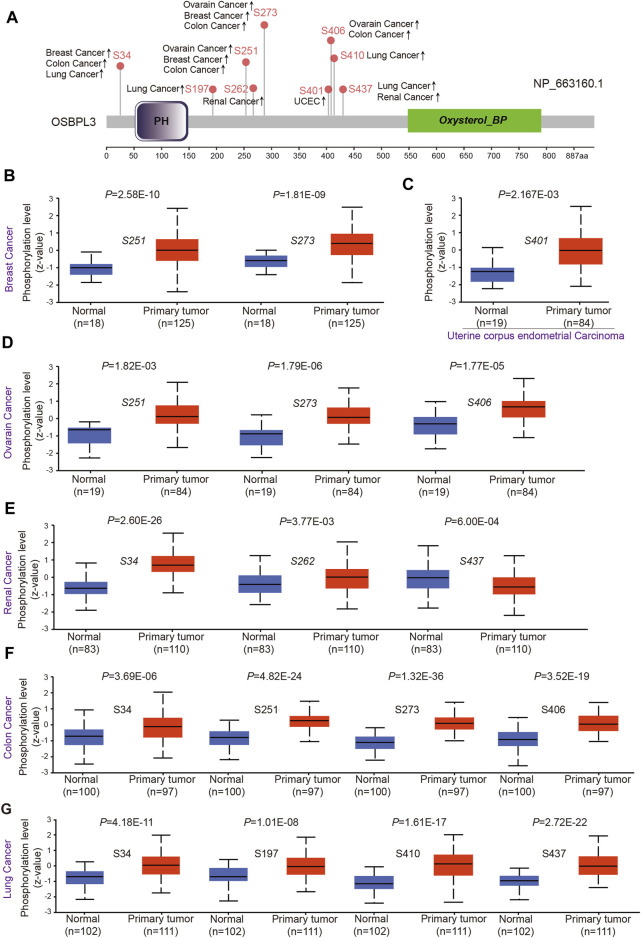
Phosphorylation analysis of OSBPL3 protein in different tumors. **(A)** Expression level of OSBPL3 phosphoprotein sites (NP_663, 160.1, S34, S197, S251, S262, S273, S401, S406, and S437 sites) between primary tumors and corresponding normal tissues via the UALCAN and CPTAC datasets, which are displayed in the schematic diagram with positive results. **(B–G)** Box plots of phosphorylation analysis of OSBPL3 for different cancers, including breast cancer **(B)**, uterine corpus endometrial carcinoma **(C)**, ovarian cancer **(D)**, renal cancer **(E**), colon cancer **(F)**, and lung cancer **(G)**.

To evaluate whether phosphorylation of OSBPL3 has an effect on tumors, we compared the differences in OSBPL3 phosphorylation levels between primary tumor and normal tissues. Figure 7A summarizes the OSBPL3 phosphorylation sites, and Figures 7B–G analyze the phosphorylation status of OSBPL3 at different sites in different tumors—breast cancer, uterine corpus endometrial carcinoma, ovarian cancer, renal cancer, colon cancer, and lung cancer. The results showed S34, S251, and S273 loci within the PH domain of OSBPL3 represent a higher phosphorylation level in all primary tumor tissues compared with corresponding normal tissues, followed by S406 and S437. We also confirmed the CPTAC-identified phosphorylation of OSBPL3 used the PhosphoNET database and found that OSBPL3 phosphorylation in the cell cycle was experimentally supported by one publication and our previous data (Table 1).

**TABLE 1:** Analysis of CPTAC-identified phosphorylation sites of OSBPL3 via the PhosphoNET database.

Site	Sequence	Experimentally confirmed^a^	Hydrophobicity	Phosphorylation site similarity score	Maximum kinase specificity	Sum kinase specificity score	Conservation score
S34	KQGSRQDSWEVVEGL	19,369,195	−1.240	−57.5	534	22,348	7.3
S197	QNLFQTGSNVSFSCG	NA	−0.280	−62.4	385	14,942	9.1
S251	DVLHRTYSAPAINAI	19,369,195	−0.167	−61.0	510	21,466	18.8
S262	INAIQGGSFESPKKE	NA	−0.920	−58.0	310	13,168	4.6
S273	SPKKEKRSHRRWRSR	NA	−3.213	−58.6	420	16,727	9.7
S410	AESLLLDSPAVAKSG	NA	0.220	−51.8	501	20,366	13.6
S437	RALVHQLSNESRLSI	19,369,195	−0.373	−56.6	381	15,792	17.6

a The PMID (PubMed Unique Identifier) information of the publication was provided.

NA, not available.

This observation merits further molecular and cellular experiments to explore the potential role of phosphorylation of OSBPL3 in tumorigenesis.

### Immune Infiltration Analysis

**FIGURE 8:**
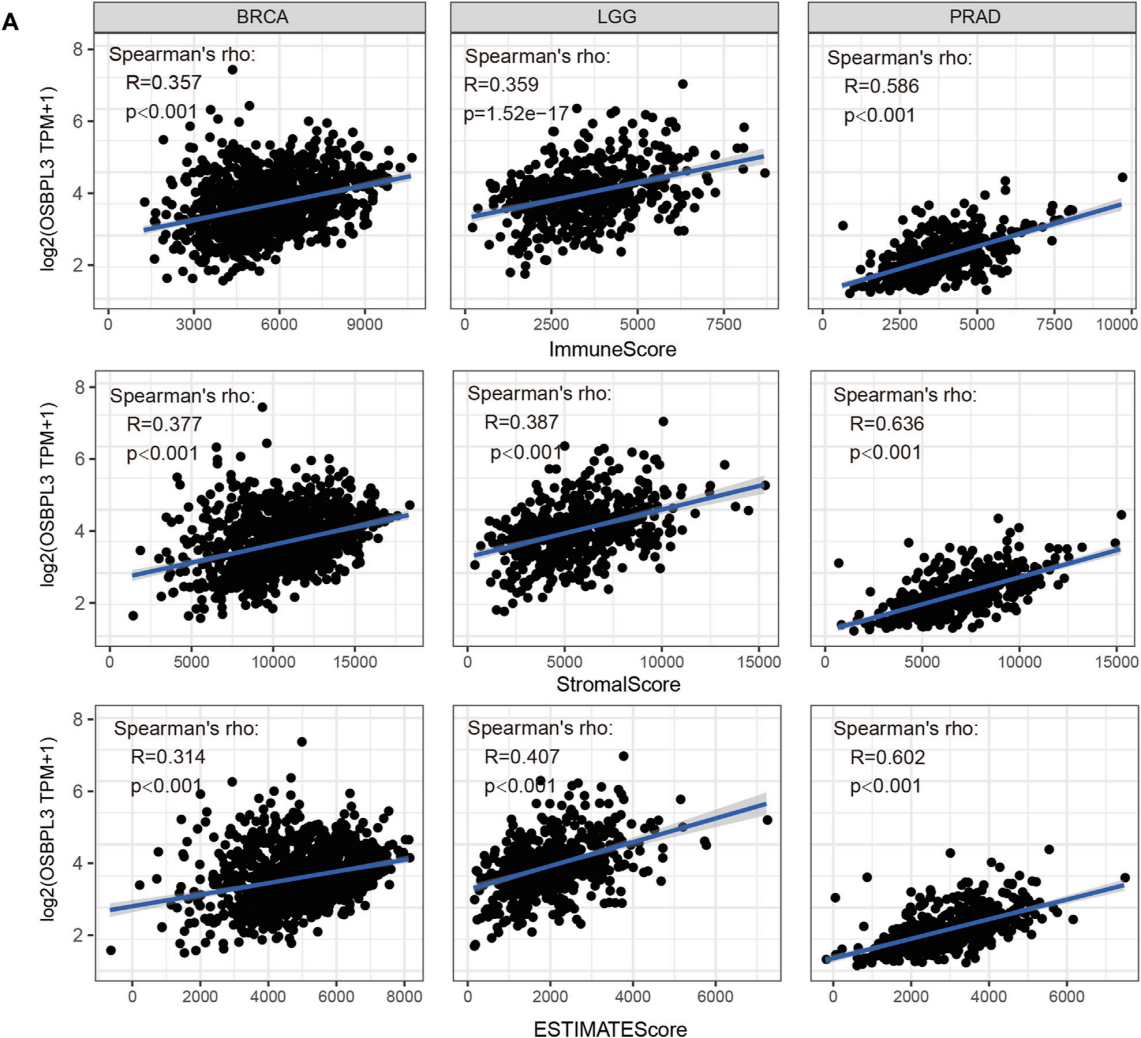
Correlation analysis between OSBPL3 expression and immune infiltration score in different tumors. We estimated the immune cells, stromal cells, and tumor purity in the tumor microenvironment based on the ESTIMATE algorithm for “Est_ImmuneScore,” “ESTIMATEScore,” and “StromalScore,” and we analyzed the relationship between them and the expression level of OSBPL3, respectively, in the top three tumors—breast cancer (BRCA), lung cancer (LGG), and prostate adenocarcinoma (PRAD)—which are most relevant.

The tumor microenvironment (TME) plays an important role in prognosis and treatment response (Jiang et al., 2020). To evaluate the effect of OSBPL3 expression level on tumor-infiltrating immune and stromal cells—the two main components of the TME closely associated with the initiation, progression, or metastasis of cancer—we performed the ESTIMATE algorithm to calculate the immune scores and stromal scores from different types of tumors (Supplementary Figures S7–9). As shown in Figure 8, OSBPL3 was significantly associated with infiltrating immune and stromal cells in the top three tumors (BRCA, LGG, PRAD) (all *p* < 0.001).

**FIGURE 9:**
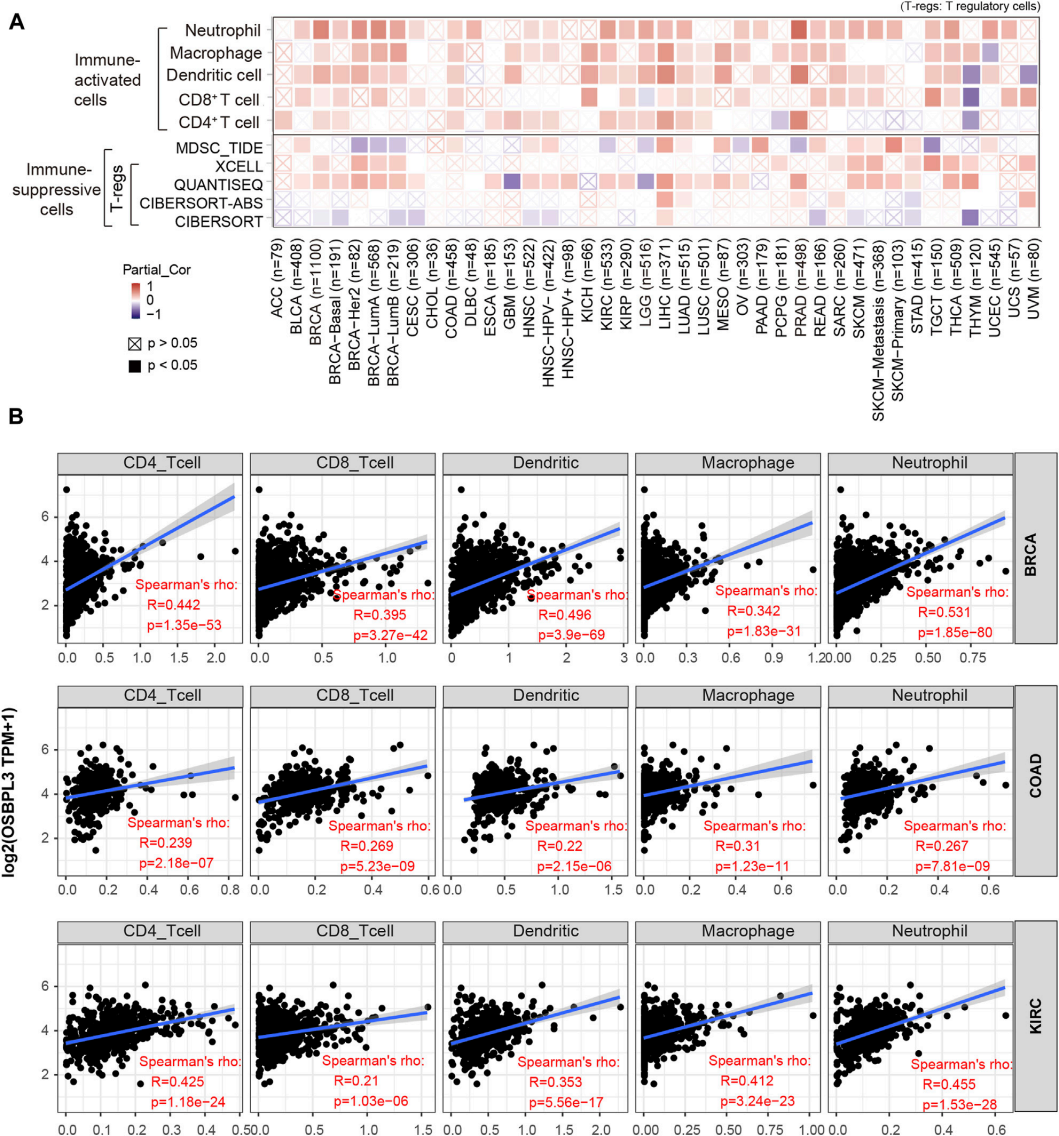
Correlation analysis between OSBPL3 expression and immune cells. **(A)** Spearman correlation analysis heatmap of infiltration level of various immune cells and OSBPL3 gene expression in diverse tumor tissues (horizontal axis: different tumor tissues; vertical axis: different immune scores; different colors represent correlation coefficients; negative values represent negative correlations. The stronger the correlation, the darker the color. The Wilcox test was performed. *p < 0.05; **p < 0.01; ***p < 0.001). **(B)** Correlation between the expression level of OSBPL3 gene and the infiltration level of CD4^+^ T cells, CD8^+^ T cells, dendritic cells, macrophages, and neutrophils in breast cancer (BRCA), colon adenocarcinoma (COAD), and kidney renal clear cell carcinoma (KIRC) (Spearman correlation analysis was performed).

Based on the characteristics of the immune cell infiltrates in the TME, the tumors were preliminarily divided into “hot” and “cold” tumors (Galon and Bruni, 2019). The “hot” tumors are infiltrated with more immune active cells (neutrophils, macrophages, CD8^+^ T cells, dendritic cells, CD4^+^ T cells, Th1), which can produce a better response to immunotherapy drugs; in contrast, the “cold” tumors with less immune cell infiltration and more proportion of immune suppressive cells (myeloid-derived suppressor cells (MDSCs), T regulatory cells (Tregs)) have a weak response to immunotherapy (Lui et al., 2020; Yang et al., 2020; Biffi and Tuveson, 2021). Thus, we explored the relationship between the OSBPL3 expression level and the active immune cells in different cancer types of TCGA. Figure 9A shows a statistical positive correlation between OSBPL3 expression and the immune active cells of neutrophils, dendritic cells, and CD8^+^ T cells in the tumors of PRAD and LIHC. The top three tumors with the strongest correlation between OSBPL3 expression and all the immune infiltration cells are BRAC, COAD, and KIRC (Figure 9B) (all *p* < 0.001). Unfortunately, OSBPL3 appears to be weakly correlated with immune suppressive cells of MDSCs and Tregs in multiple tumors, which suggests that patients with high expression of OSBPL3 are more likely to have the “hot” tumors in the TME and might have a better immunotherapeutic response.

**FIGURE 10:**
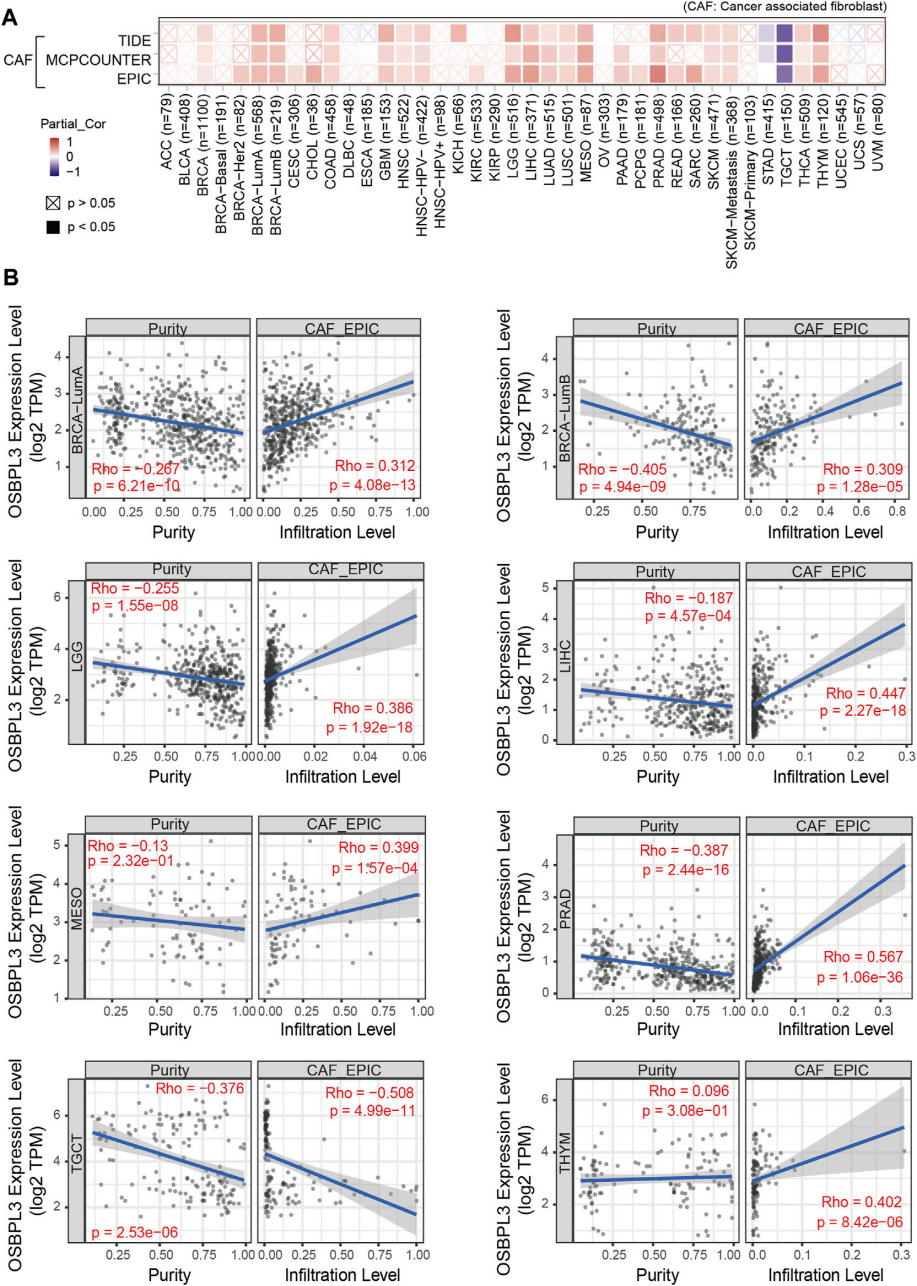
Correlation analysis between OSBPL3 expression and infiltration of cancer-associated fibroblasts (CAFs). **(A)** Heatmaps of infiltration level of OSBPL3 gene expression and CAFs in multiple tumor tissues (horizontal axis: different tumor tissues; vertical axis: different immune scores; different colors represent correlation coefficients. The stronger the correlation, the darker the color. The Wilcox test was performed. *p < 0.05; **p < 0.01; ***p < 0.001). **(B)** Correlation between the expression level of OSBPL3 gene and the infiltration level of CAFs in BRCA-luminal A, BRCA-luminal B, LGG, LIHC, MESO, PRAD, TGCT, and THYM (Spearman correlation analysis was performed).

Additionally, cancer-associated fibroblasts (CAFs) play a role in regulating the interaction between immune effector cells and cancer cells and are associated with tumor progression and poor prognosis (Desbois and Wang, 2021). Thus, we explored the relationship of OSBPL3 with CAFs, and the results showed a positive correlation between the infiltration level of CAFs and OSBPL3 expression in the tumors of BRCA-luminal A, BRCA-luminal B, LGG, LIHC, MESO, PRAD, TGCT, and THYM (Figures 10A,B) while a negative correlation in TGCT.

**FIGURE 11:**
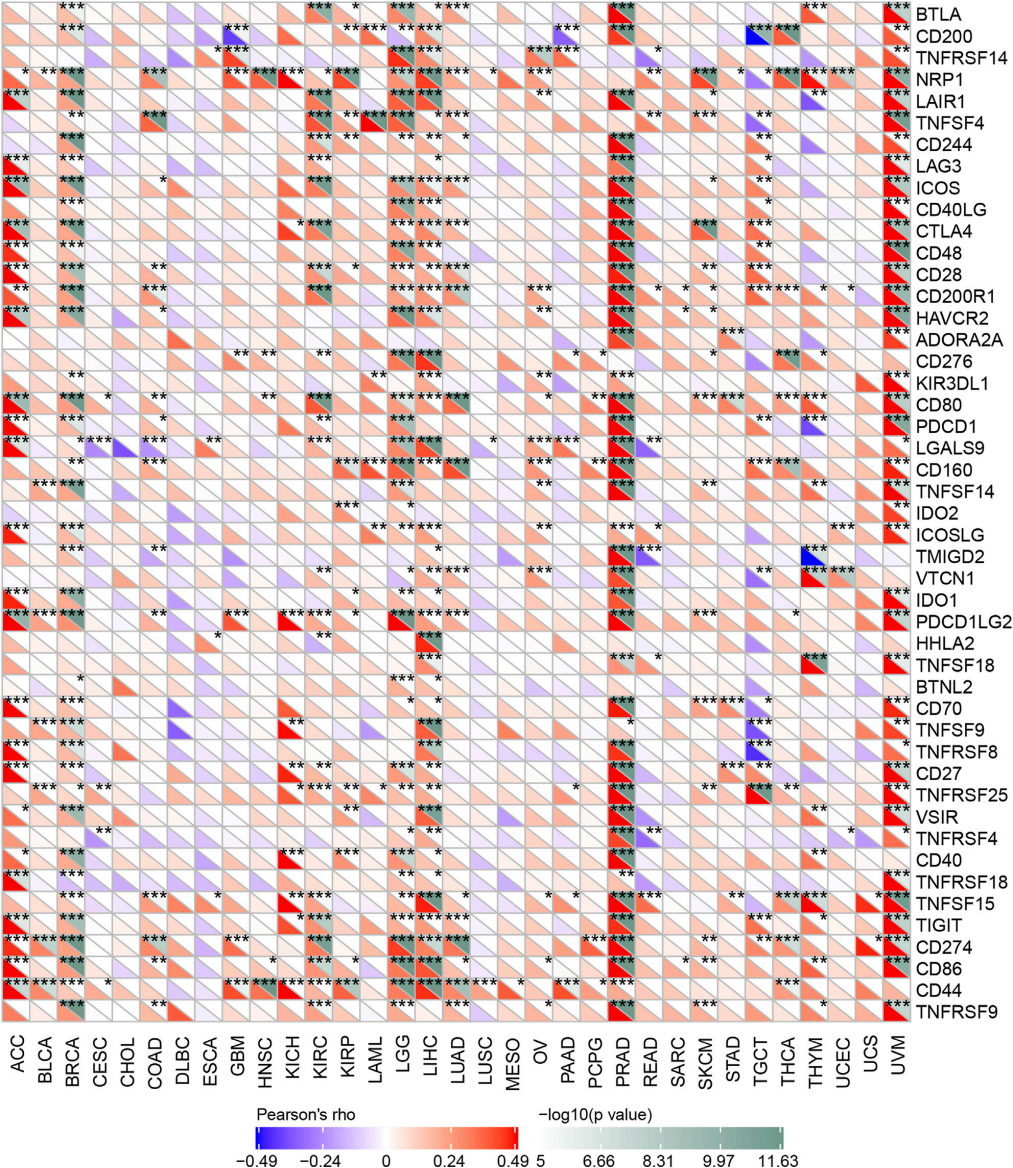
Correlation analysis between OSBPL3 expression and immune checkpoints. Heatmaps of the immune checkpoint–related genes with OSBPL3 expression in different tumor tissues (horizontal axis: different immune checkpoint genes; vertical axis: different tumor tissues. Each box represents the correlation between the expression of the immune checkpoint gene and the OSBPL3 gene in corresponding tumors, and different colors represent changes in correlation coefficients. *p < 0.05; **p < 0.01; ***p < 0.001).

Immune checkpoint inhibitors (ICIs) have affected the therapeutic landscape for a variety of tumors, but biomarkers associated with ICIS efficacy are still lacking (Bagchi et al., 2021). To analyze the relationship between OSBPL3 and immune checkpoint genes, we extracted and calculated more than 40 common immune checkpoint genes in diverse cancer types of TCGA (Figure 11). The results showed over 30 immune checkpoint genes were strongly associated with OSBPL3 expression in UVM, PRAD, and ACC tumors, and then in LIHC, LGG, and BRAC, which are the ones that are positively associated with OSBPL3 prognosis.

These results indicated that tumors with high expression of OSBPL3 are infiltrated with more immune active cells and seem to represent “hot” tumors and more immune checkpoint genes, which may play a potential role in response to the immune microenvironment and may benefit from immune therapeutic interventions.

### Enrichment and Molecular Mechanism Analysis of OSBPL3-Related Partners

**FIGURE 12:**
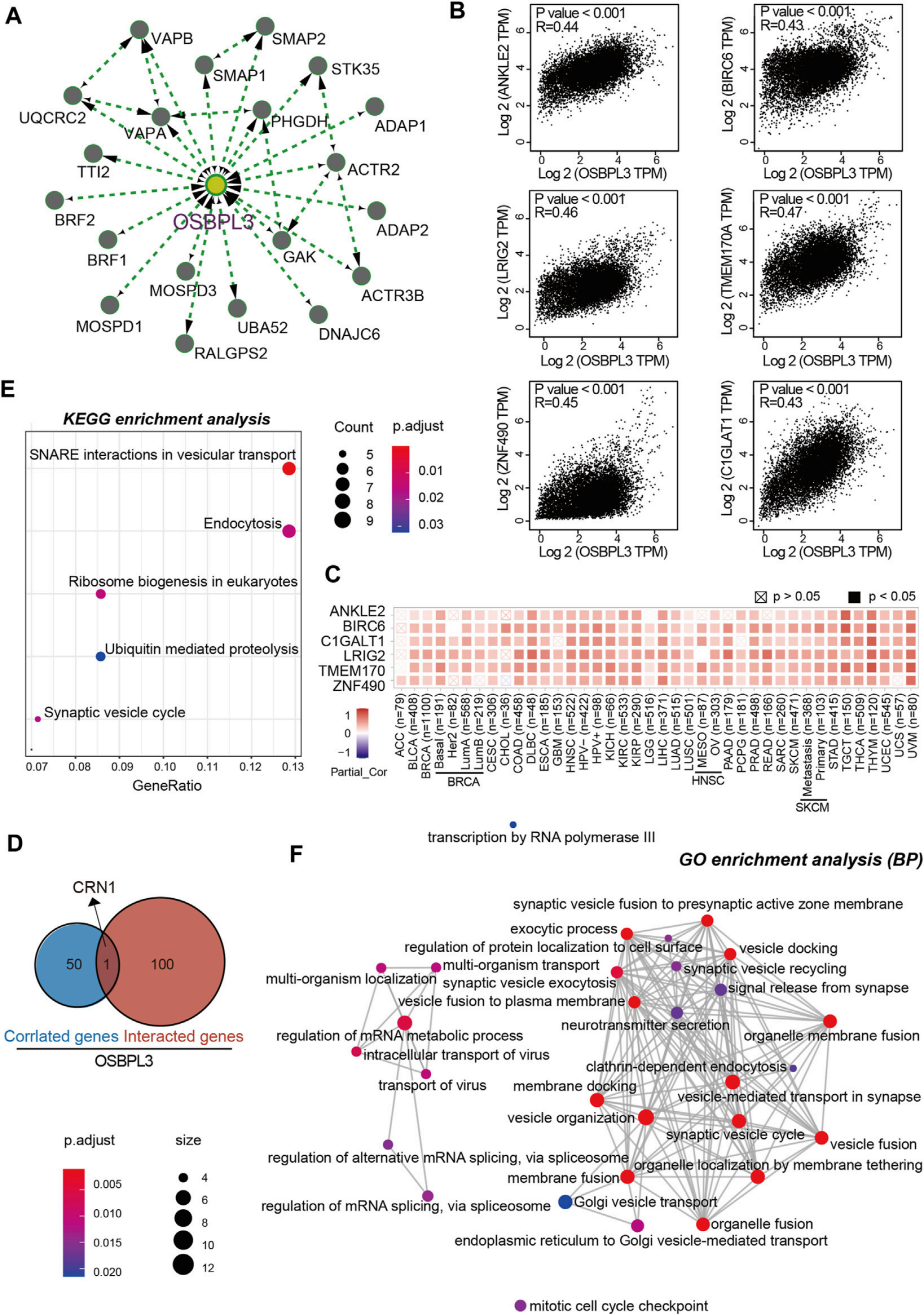
OSBPL3-related gene enrichment analysis. **(A)** The top 20 available experimentally determined OSBPL3-binding proteins using the STRING tool. **(B)** The top 100 OSBPL3-correlated genes in TCGA projects on GEPIA2 and expression correlation between OSBPL3 and top six genes, including ANKLE2, BIRO6, LRIG2, TMEM170A, ZNF490, and C1GLAT1. **(C)** Heatmap of correlation between OSBPL3 and top six genes in different cancer types. **(D)** An intersection analysis of OSBPL3-binding and -correlated genes. **(E–F)** KEGG pathway analysis **(E)** and GO enrichment analysis-biological process (BP) **(F**) of the OSBPL3-binding and -interacted genes were performed.

To further investigate the molecular mechanism and function of the OSBPL3 gene in tumorigenesis, we screened out the OSBPL3-binding proteins and correlated genes by a series of pathway enrichment analyses. Figure 12A shows the interaction network of top 20 OSBPL3-binding proteins, and Figure 12B shows the top six of the top 100 positively correlated genes with the OSBPL3 expression level—ANKLE2 (*R* = 0.44), BIRC6, LRIG2, TMEM170A, ZNF490, and C1GLAT1 genes (all *p* < 0.001). Similar results were found in the majority of cancers by a corresponding heatmap (Figure 12C). An intersection analysis of the above two groups reached one common member, namely, CRN1 (Figure 12D).

**FIGURE 13:**
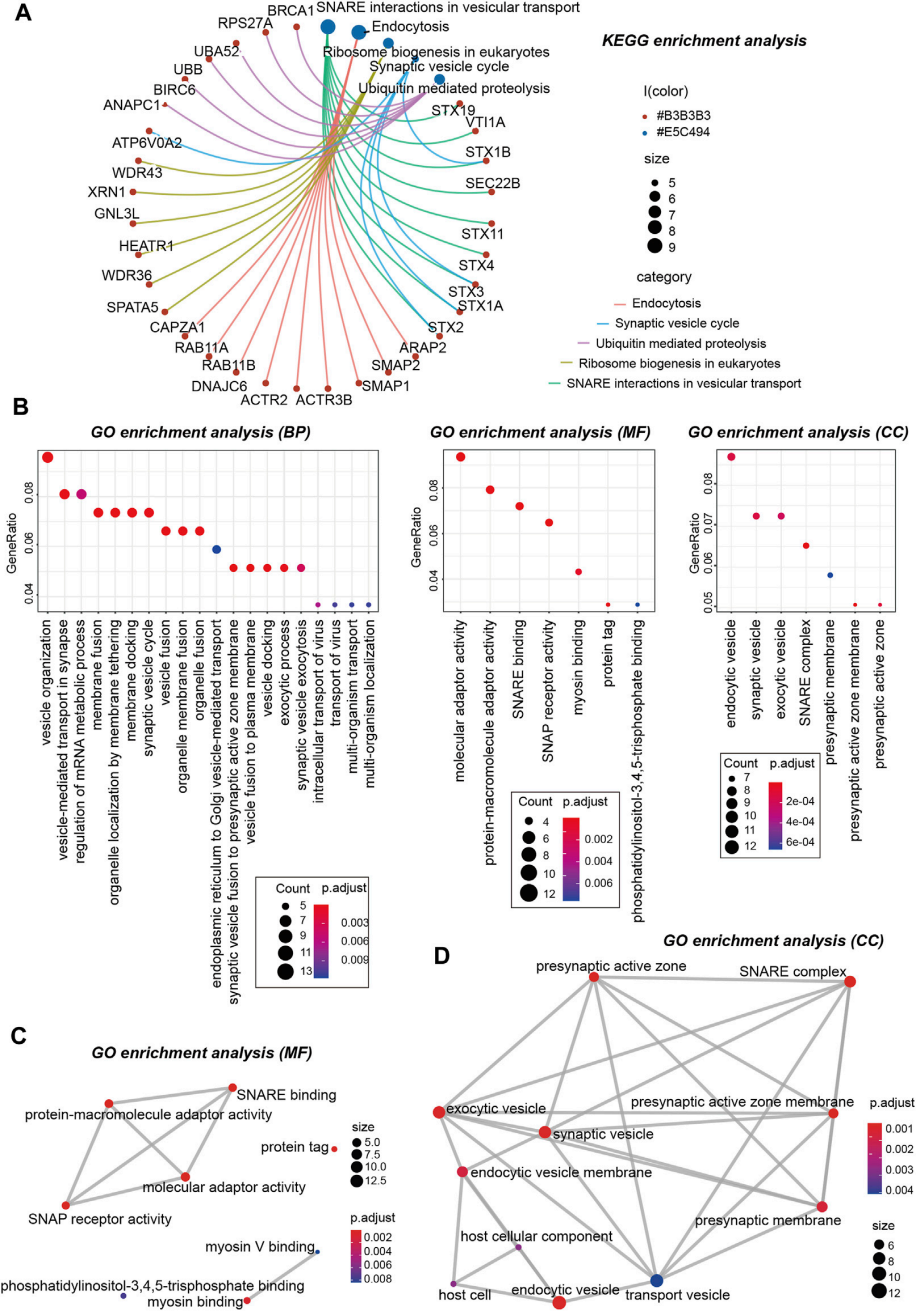
KEGG and GO analyses-biological process (BP)/cellular component (CC)/molecular function (MF) of OSBPL3-related genes in tumors. **(A)** cnetplot for the KEGG analysis. **(B)** Bubble diagram of GO enrichment analysis. **(C–D)** GO enrichment analysis-MF **(C)** and CC **(D)** were performed.

Next, we performed KEGG and GO enrichment analyses. The KEGG enrichment analysis of Figures 12E, 13A suggests that “SNARE interactions in vesicular transport,” “Endocytosis,” “Synaptic vesicle cycle,” and “Ribosome biogenesis in eukaryotes” might be involved in the molecular mechanism of OSBPL3 on tumor pathogenesis. The GO enrichment analysis—biological process (BP), molecular function (MF), and cellular component (CC)—further showed that most of the related genes are associated with the pathways or cellular biology of mRNA metabolism, synaptic/endocytic/exocytic vesicles, SNARE binding, protein molecular adaptor activity, membrane fusion/tethering/docking, and others (Figures 12F, 13B–D–D; Supplementary Figure S10).

Taken together, these data suggest the biological processes and molecular mechanism in which OSBPL3 may be involved and further investigations are needed for exploration and confirmation.

## Discussion

This is the first systematic pan-cancer analysis, to our knowledge, to comprehensively summarize the molecular features, clinical prognosis, and mechanisms characterizing the presentation of the OSBPL3 gene and its impact on the process of tumorigenesis in a total of 33 different tumors. We show a general high level of OSBPL3 expression in major cancers compared with normal tissues from multiple databases and cancer biopsies. A high abundance of intra-tumoral OSBPL3 cells had prolonged survival that are seen in a significant proportion of cancer patients, and it seems likely that the mutation, phosphorylation, immune infiltration, and cell membrane pathways of OSBPL3 affect the disease forward. On the basis of this analysis and the previous work, it is clear that OSBPL3 plays an important role in the process of tumorigenesis.

Although there are few studies on the expression and role of OSBPL3 in tumors, our study shows that OSBPL3 was highly expressed in multiple human tumors compared with normal tissues from both the detection of the TCGA database and experiments of tissues of tumor patients. Nevertheless, the survival outcome data for OSBPL3 show distinct conclusions for different tumors. In this work, a group of survival analyses by GEPIA2, OncoLnc tools, and Kaplan–Meier plotter indicated a strong prognostic relevance between high level of OSBPL3 expression and poor OS, DFS, PFI, and DFI prognosis for low-grade glioma; poor OS, DFS, and PFI for uveal melanoma; and poor PFI and DFI for prostate adenocarcinoma while better clinical prognosis for testicular germ cell tumor cases. Xu et al. reported that downregulation of OSBPL3 correlates with reduced survival of colon cancer patients with advanced nodal metastasis and grade 3 colon cancer (Xu et al., 2020), but another recent research showed the contrary result that upregulation of OSBPL3 by HIF1A promotes colorectal cancer progression (Jiao et al., 2020). Interestingly, our experimental data showed that the higher the grade of colon cancer, the higher the level of OSBPL3 expression. Thus, the current clinical evidence still cannot support the role of OSBPL3 expression with the clinical outcome in different cancers, and more sample sizes are needed for exploration and confirmation.

In this work, we integrated the alterations of the OSBPL3 gene, including splice, mutations, and its prognosis data. Komor et al. reported that the spliced region of OSBPL3 exon 9 is one of the cancer-specific aberrant splicing biomarkers in colorectal cancer cells (Komor et al., 2017), and we also found the X676_splice/V676G alteration in the oxysterol domain. Additionally, we show that the greater the number of mutations present within an individual patient, the worse the outcome in skin cutaneous melanoma. Lefebvre et al. reported that OSBPL3 was more frequently mutated in metastatic breast cancer—and associated with poor outcomes—as compared to early breast cancer (Lefebvre et al., 2016), and Njeru et al. suggested the mutations of OSBPL3 contribute to carcinogenesis involving the deregulation of various molecular processes such as lipid metabolism, proliferation, and cell survival (Njeru et al., 2020). We also first presented evidence that the tumor with high expression of OSBPL3 had higher TMB, which is one of the important prediction markers of the efficacy of tumor immunotherapy. These data will be helpful for future research on the mechanism and therapeutic application of OSBPL3 in cancers.

Notably, the total protein and phosphorylation of OSBPL3 at the S426, S251, and S273 loci within the pleckstrin homology (PH) domain were both at elevated levels. Lehto et al. reported that the PH domain of OSBLP3 binds the phosphoinositide-3-kinase (PI3K) products by an FFAT motif (EFFDAxE) (Lehto et al., 2005), and Gulyas et al. showed that ORP3 phosphorylation regulates phosphatidylinositol-4-phosphate and Ca (2+) dynamics (Gulyas et al., 2020). More experimental evidence is fully needed to determine how the phosphorylation of OSBPL3 affects the initiation and progress of tumors.

We also first integrated the correlation between OSBPL3 expression and the tumor microenvironment including immune infiltration and stromal cells, immune-related cells, and immune checkpoint inhibitors (ICIs) in a variety of tumors. We found that high expression of OSBPL3 is infiltrated with more immune active cells and seems to represent “hot” tumors and more immune checkpoint genes, which may play a potential role in response to the immune microenvironment and may benefit from immune therapeutic interventions. Although the role and interaction between OSBPL3 and immune infiltration need more in-depth molecular experimental verification, our large sample analysis and prediction of cancer database validation play a positive and hint role. Interestingly, based on the BELOB trial, the expression of OSBPL3 is significantly associated with treatment response of bevacizumab and CCNU chemotherapy in recurrent GBM patients (Taal et al., 2014). In the further, we may be able to predict prognosis by detecting OSBPL3 expression in tumors. And our laboratory is also trying to further explore the application of targeted OSBPL3 therapy.

In addition, the “HomoloGene” and phylogenetic tree indicated the conservation of the OSBPL3 gene structure across diverse species, suggesting that similar mechanisms might exist under normal physiological conditions (Lopez-Guerra et al., 2008; Song et al., 2012; Zhou et al., 2012). Our analysis of the reproductive system tumors agreed with the finding of previous reports (Weber-Boyvat et al., 2015; Darbyson and Ngsee, 2016; Stein et al., 2017; Santos et al., 2018; Xue et al., 2019) but also suggested the existence of specific mechanisms, including synaptic/endocytic/exocytic vesicles, mRNA metabolism, SNARE binding, and protein molecular adaptor activity. Whether OSBPL3 plays a similar role in tumorigenesis through certain molecular mechanisms needs further verification and exploration.

There are still some limitations in our study. First, this work mostly focused on the bioinformatic analysis of the expression and potential molecular mechanisms of OSBPL3 without more experiments to explore the phenotype and function *in vitro* and *in vivo*. Our lab is focusing and trying to clarify the role of OSBPL3 at both cellular and molecular levels in different types of cancers. Second, although the ratio of immune components in the TME is significantly associated with OSBPL3, it was unable to determine whether and how OSBPL3 influences patient survival through immune infiltration. Future experimental studies may provide additional mechanistic insights into immune cell infiltration.

In summary, our studies have demonstrated that OSBPL3 plays an important role in tumorigenesis from the perspective of public databases and clinical tumor samples and is potentially a novel and specific target for cancers, which comprehensively provides insights for further investigation.

## Conclusion

In conclusion, we first identified a comprehensive analysis of OSBPL3 based on the TCGA project and summarized the molecular features, clinical prognosis, and mechanisms characterizing the presentation of the OSBPL3 gene and its impact on the process of tumorigenesis in a total of 33 different tumors, which provide a comprehensive understanding of OSBPL3 in oncogenesis and will be valuable for further in-depth research.

## Data Availability

The original contributions presented in the study are included in the article/Supplementary Material, and further inquiries can be directed to the corresponding authors.
