# Supplementary figures and images for: Clinical Value and Potential Mechanisms of Oxysterol-Binding Protein Like 3 (OSBPL3) in Human Tumors
Source: Front Mol Biosci. 2021 Oct 19;8:739978. doi: 10.3389/fmolb.2021.739978 (PMC8560696; doi:10.3389/fmolb.2021.739978)

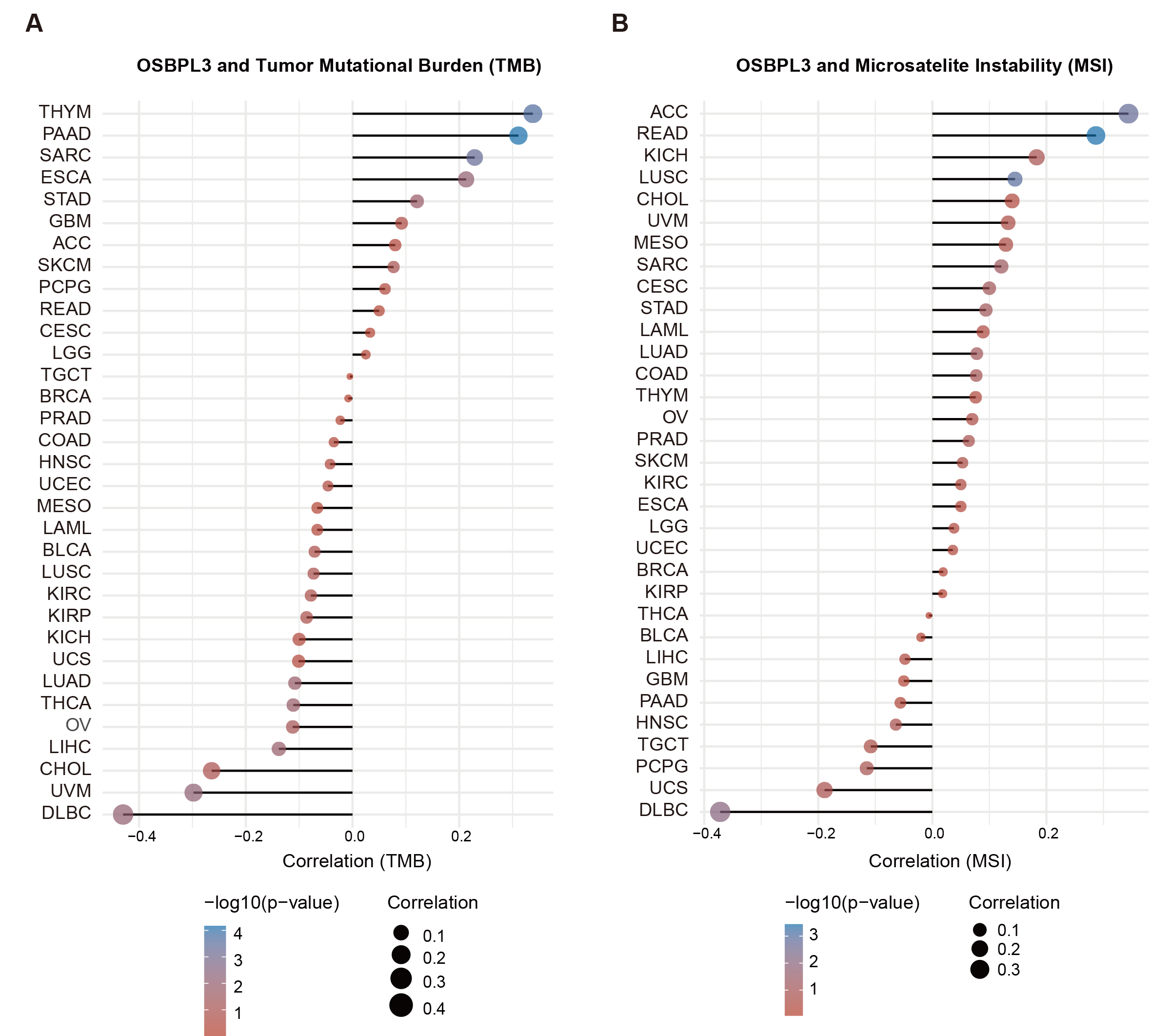

Supplement: Supplementary file 2 [file Image6.TIF]

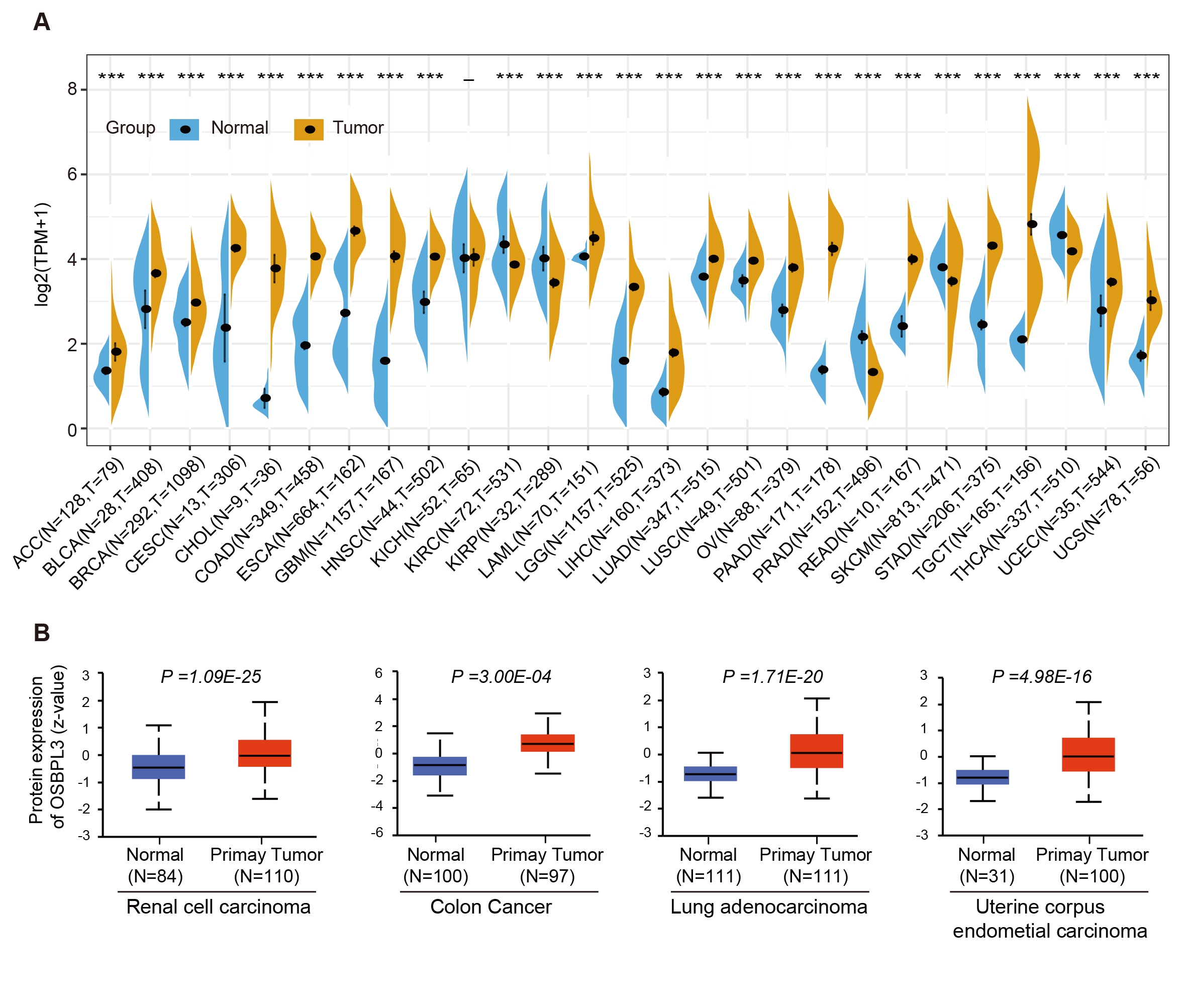

Supplement: Supplementary file 3 [file Image3.TIF]

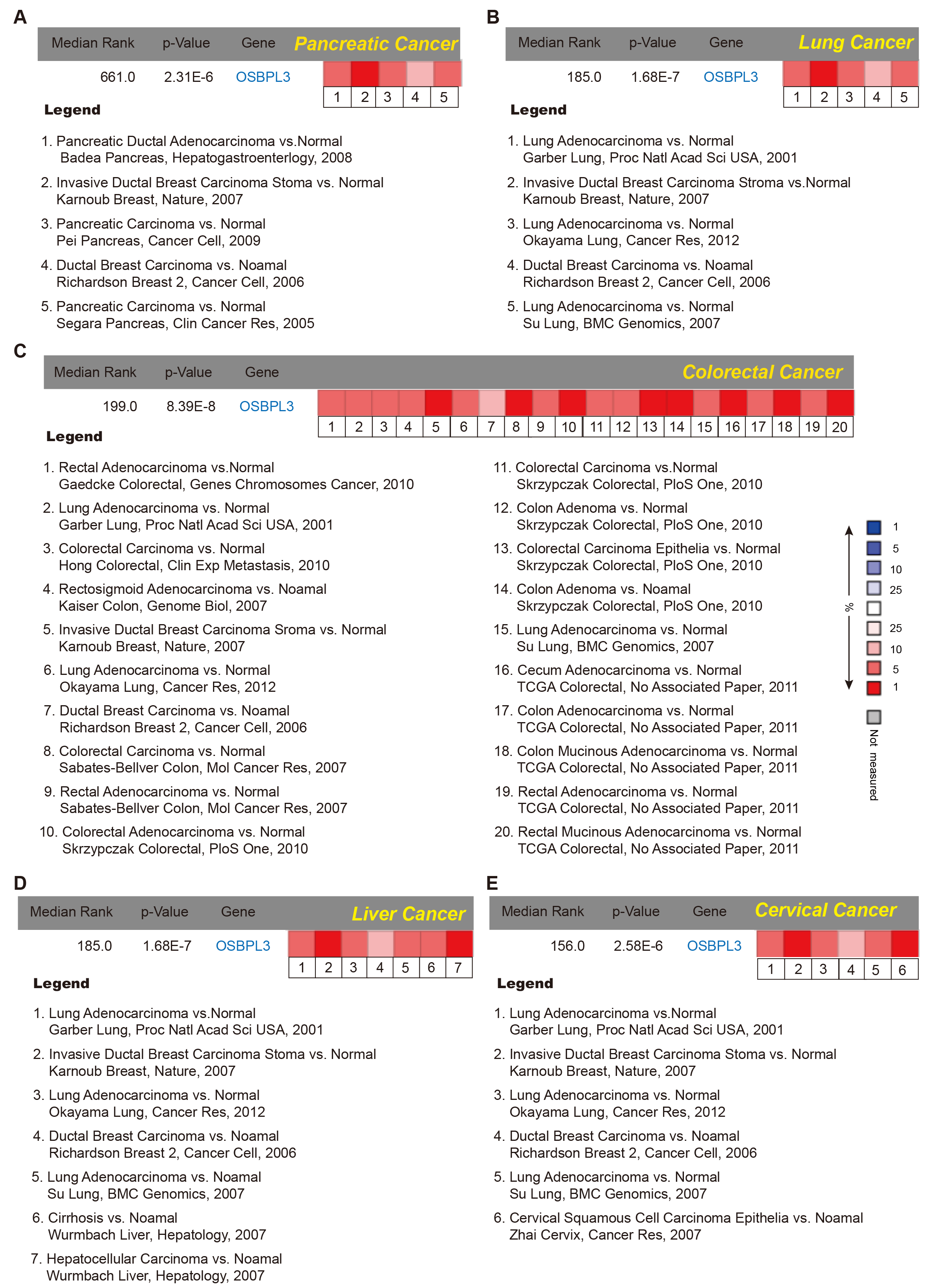

Supplement: Supplementary file 4 [file Image4.TIF]

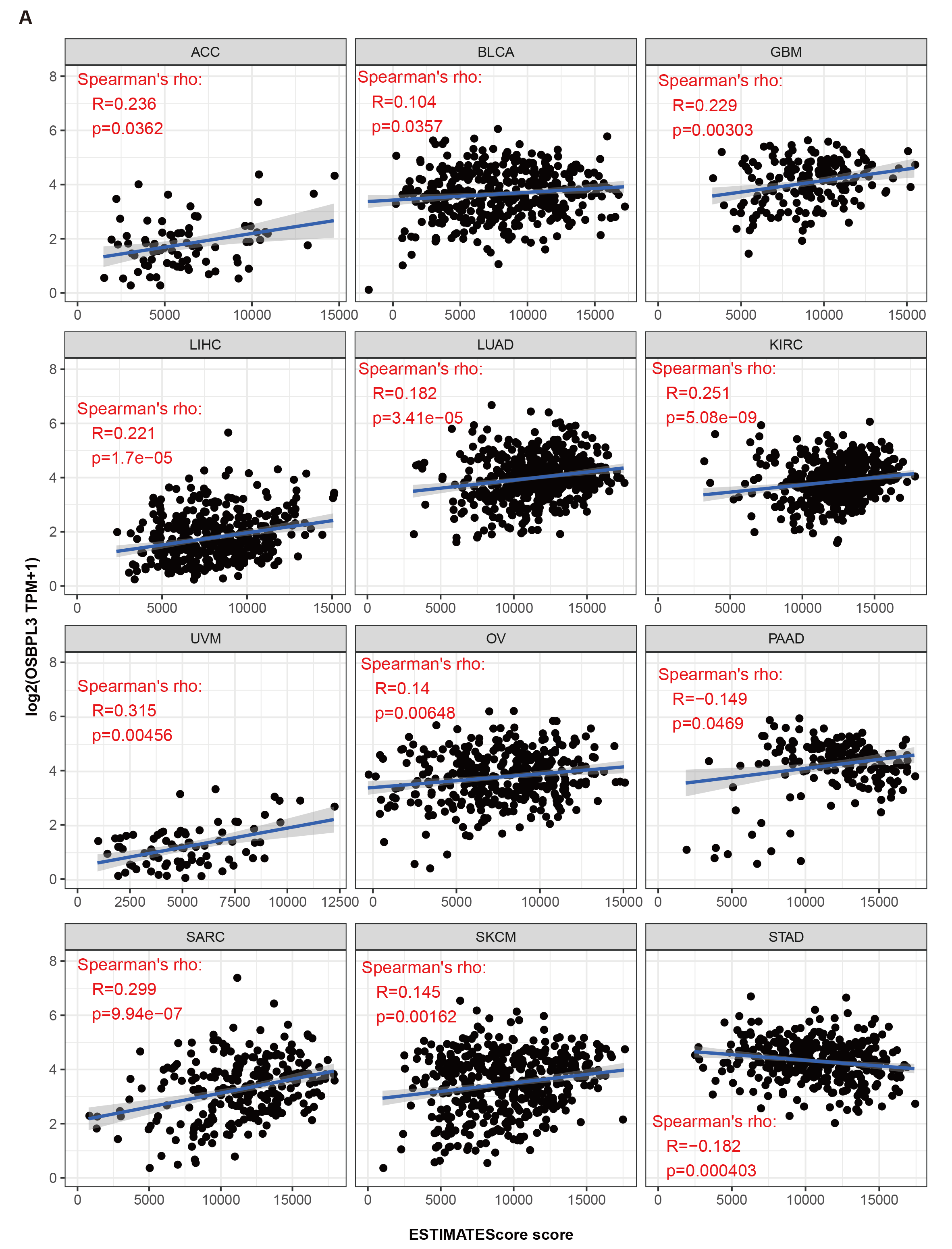

Supplement: Supplementary file 5 [file Image9.TIF]

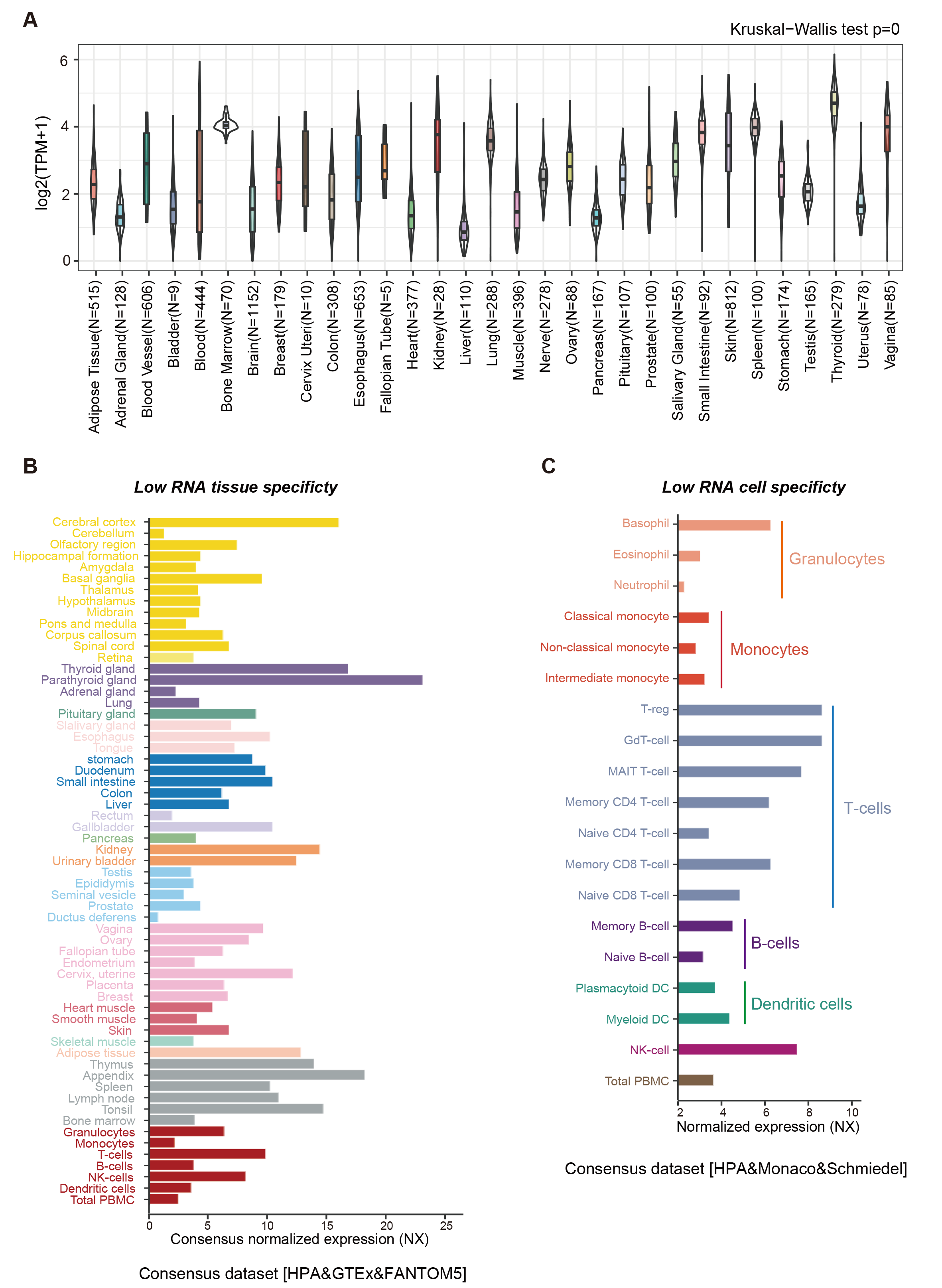

Supplement: Supplementary file 6 [file Image2.TIF]

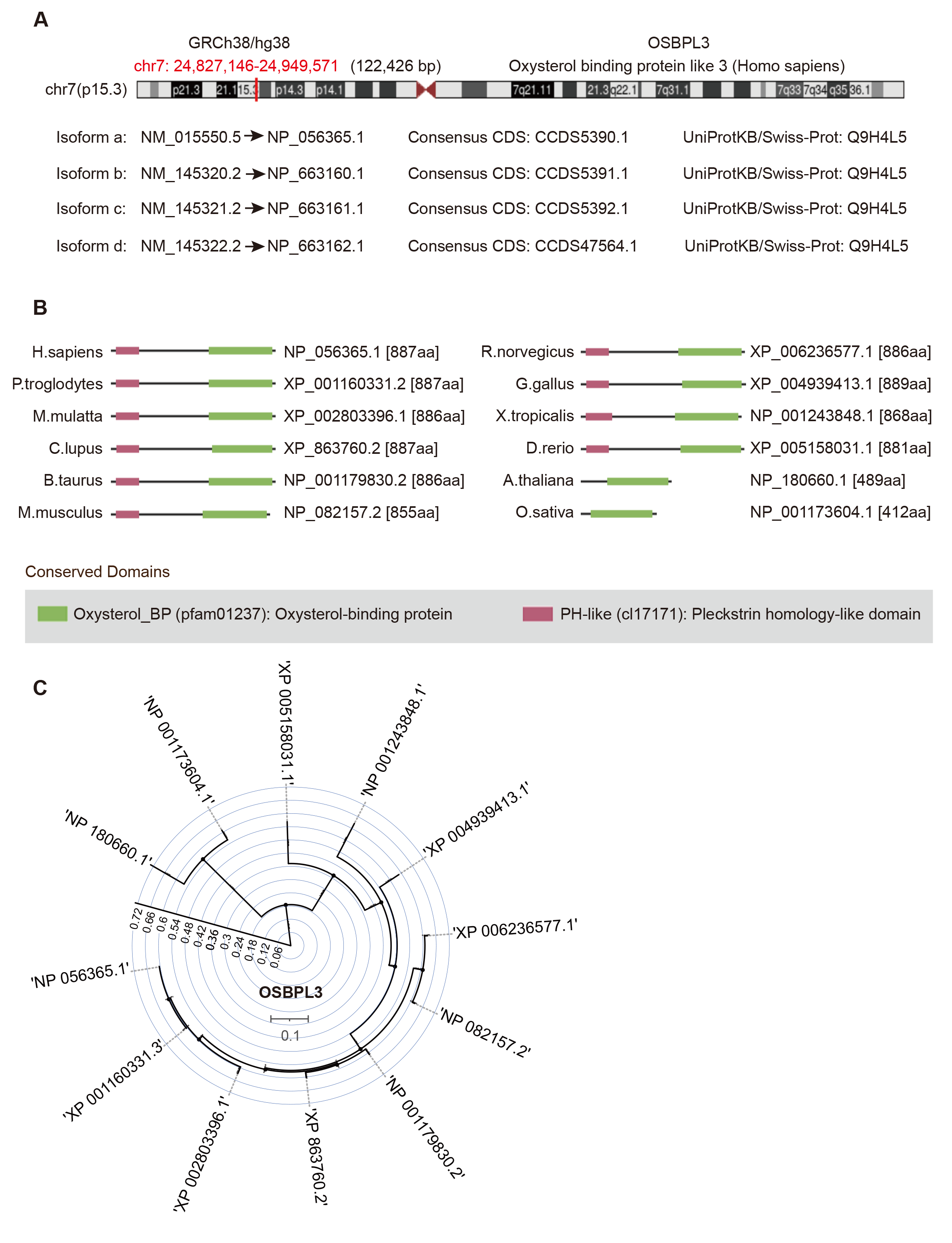

Supplement: Supplementary file 7 [file Image1.TIF]

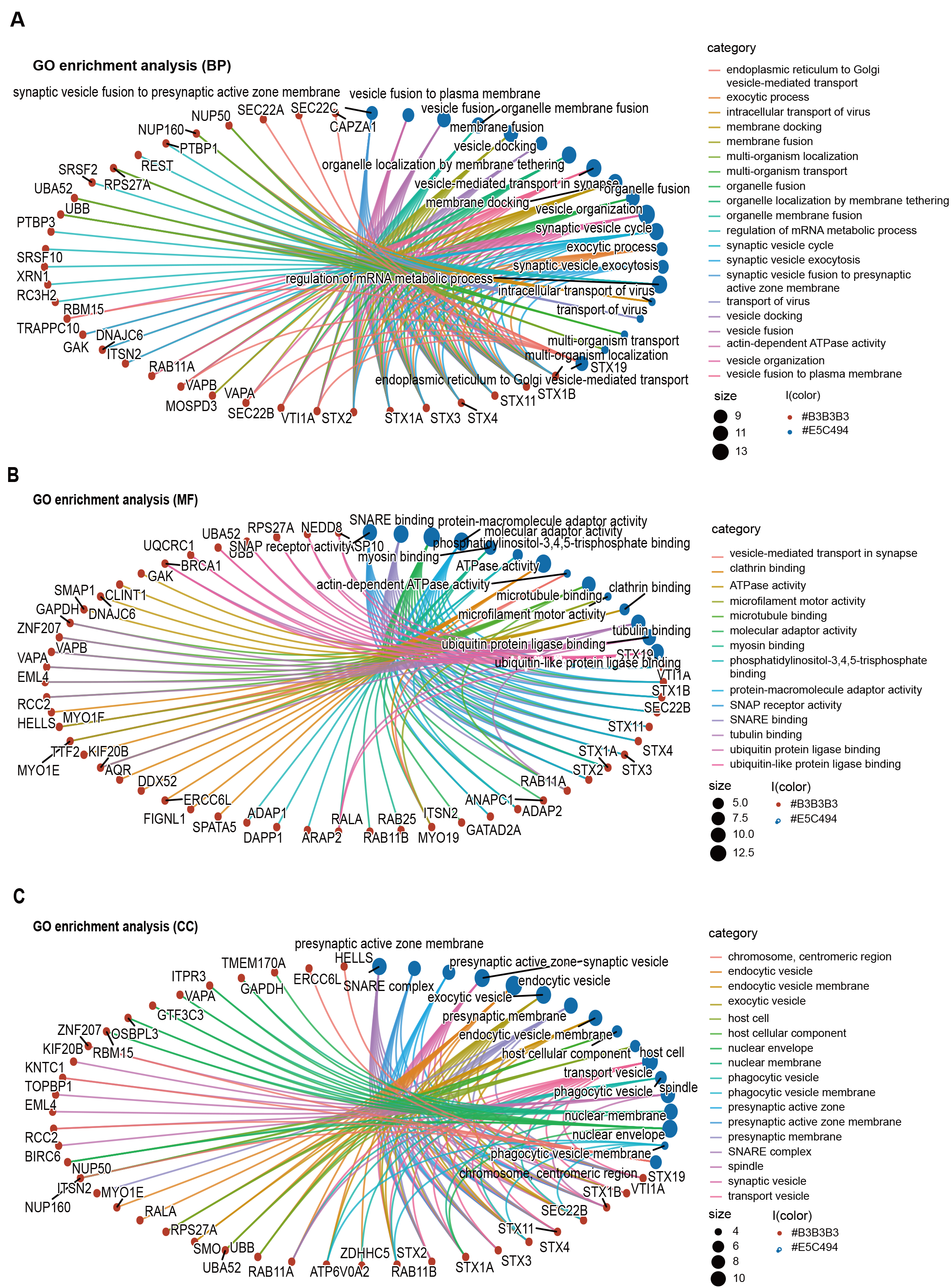

Supplement: Supplementary file 8 [file Image10.TIF]

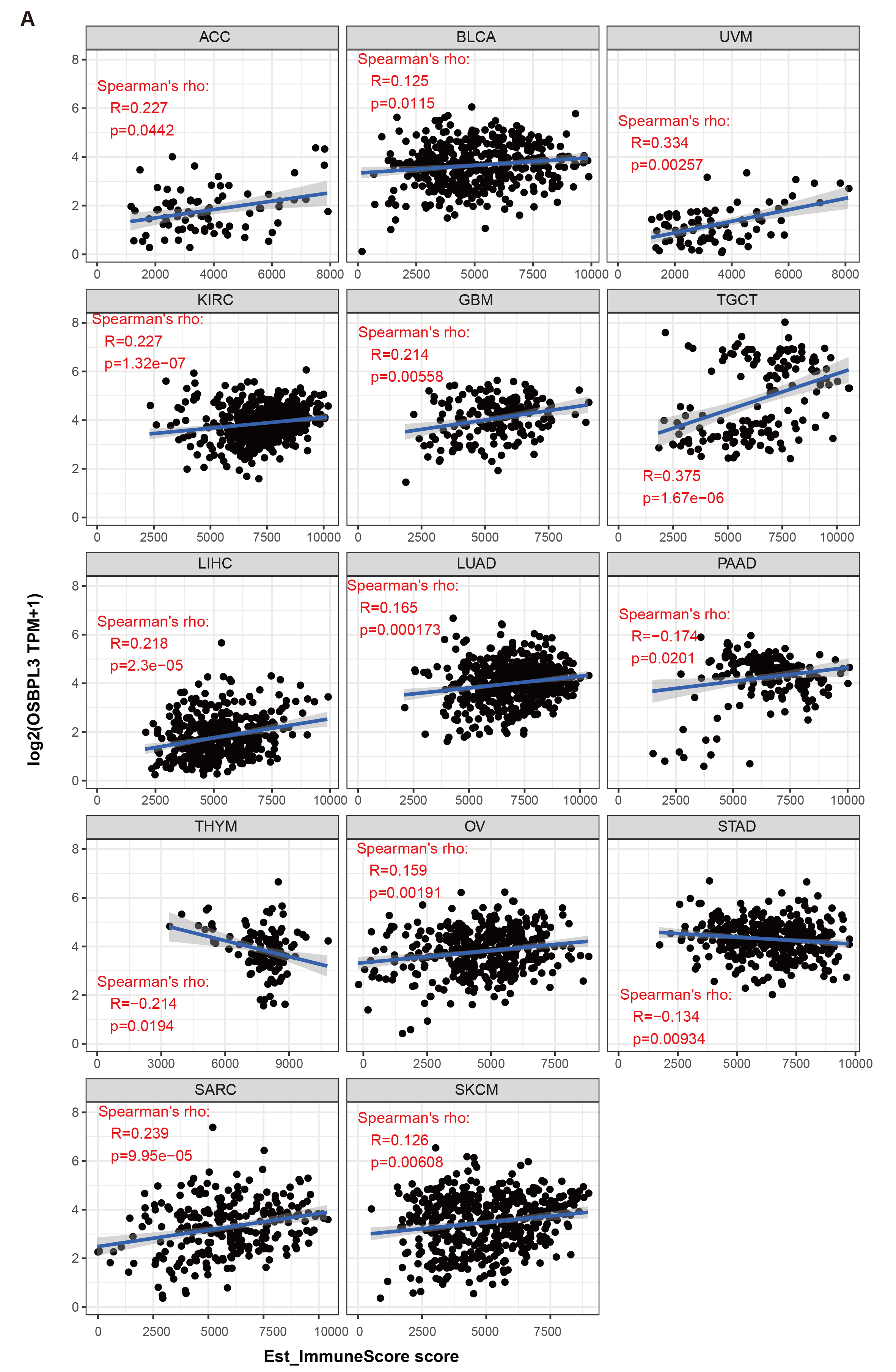

Supplement: Supplementary file 9 [file Image7.TIF]

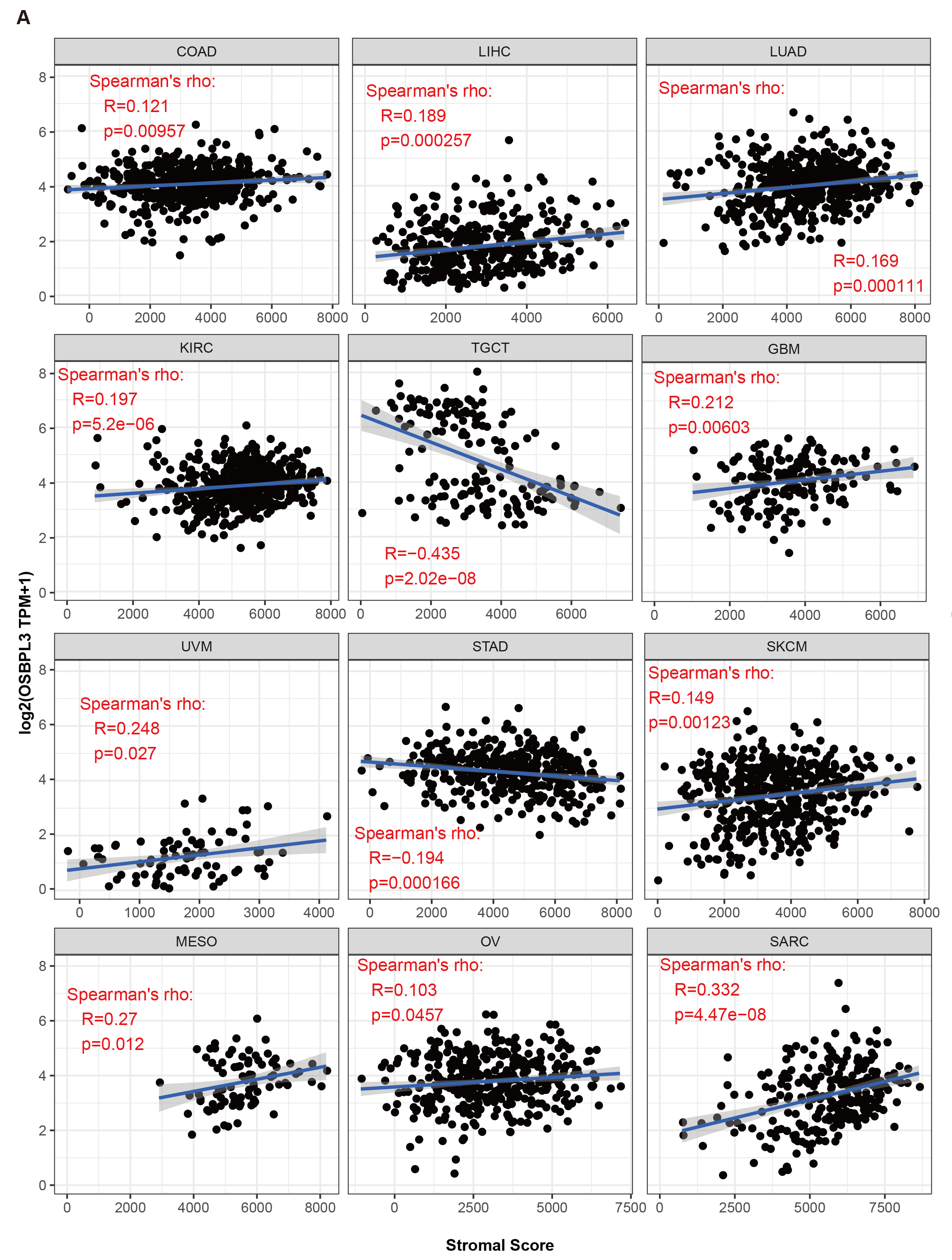

Supplement: Supplementary file 10 [file Image8.TIF]

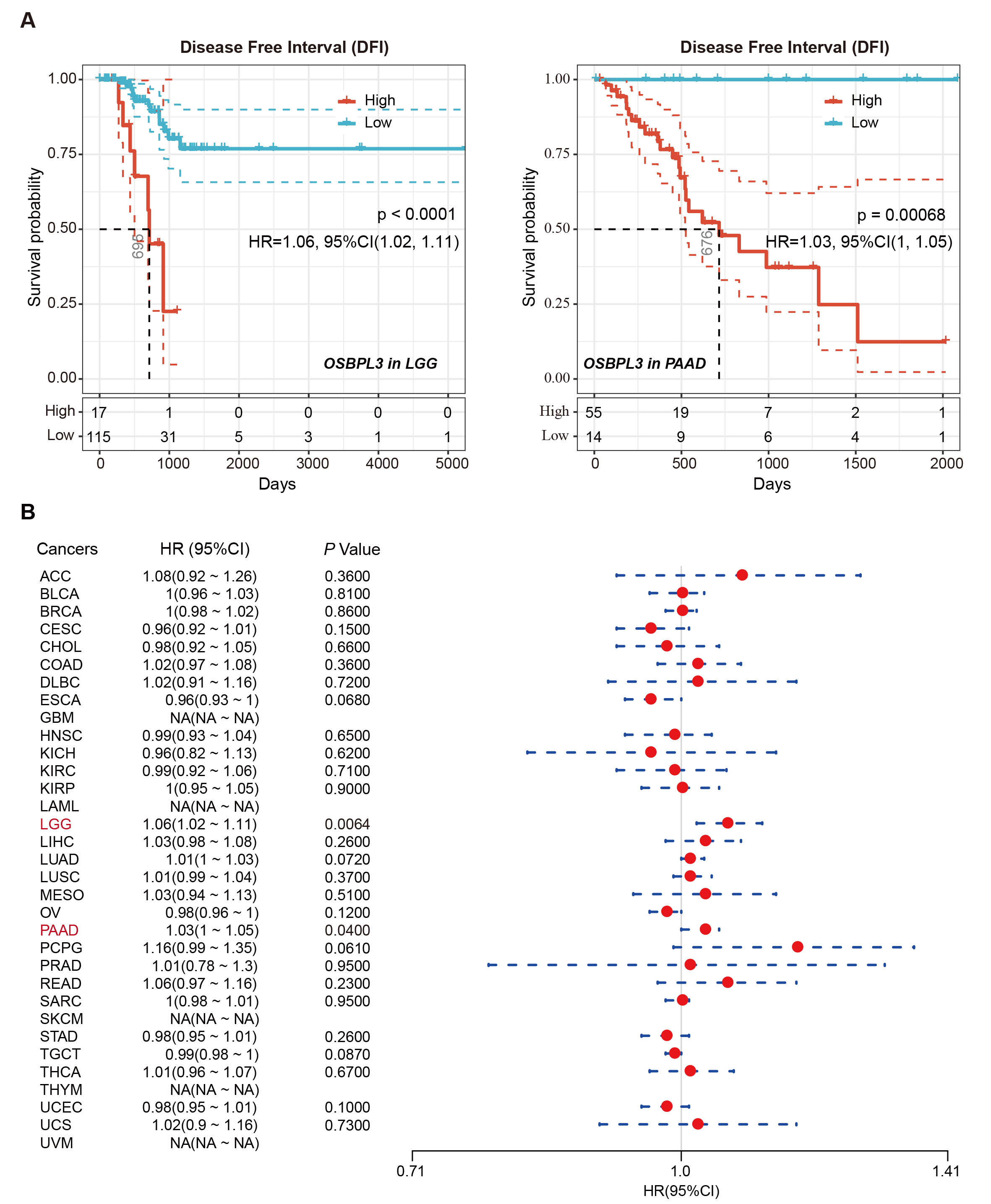

Supplement: Supplementary file 11 [file Image5.TIF]
