# Supplementary material for: Clinical Value and Potential Mechanisms of Oxysterol-Binding Protein Like 3 (OSBPL3) in Human Tumors
Source: Front Mol Biosci. 2021 Oct 19;8:739978. doi: 10.3389/fmolb.2021.739978 (PMC8560696; doi:10.3389/fmolb.2021.739978)
Supplement: Supplementary file 12 [file DataSheet1.DOCX]

Supplementary Material

# Materials and Methods

**Gene mapping and protein structure analysis**

Based on the UCSC genome browser on human Dec. 2013 (GRCh38/hg38) assembly (<http://genome.ucsc.edu/>) [1], the genome location information of the OSBPL3 gene was obtained. We also applied the "HomoloGene" function (<https://www.ncbi.nlm.nih.gov/homologene/>) of the NCBI (National Center for Biotechnology Information) to conduct conserved functional domain analysis of the OSBPL3 protein in different species. Additionally, we obtained the phylogenetic tree of OSBPL3 in different species using the constraint-based multiple alignment on-line tool of the NCBI (<https://www.ncbi.nlm.nih.gov/tools/cobalt/>).

**Gene and protein expression analysis**

We first logged into the online HPA (Human protein atlas) database (<https://www.proteinatlas.org/humanproteome/pathology>) and obtained the expression data of the OSBPL3 gene in different cells and tissues under physiological conditions by entering the word “OSBPL3”. The expression level of the OSBPL3 protein in a plasma sample, as determined via mass spectrometry-based proteomics, cancer tissues and cancer cells were estimated in the HPA database. “Low specificity” was defined by “NX (Normalized expression) ≥1 in at least one tissue/region/cell type but not elevated in any tissue/region/cell type”. The detailed information can be found at the link <https://www.proteinatlas.org/ENSG00000070882-OSBPL3>.

The UALCAN portal (http://ualcan.path.uab.edu/analysis-prot.html), an interactive web resource for analyzing cancer Omics data, allowed us to conduct protein expression analysis of the CPTAC (Clinical proteomic tumor analysis consortium) dataset.

We also logged into the Oncomine database and obtained a series of pooling analyses across at least ten comparisons were performed. The median rank for OSBPL3 across each of the analyses, the *P*-value for the median-ranked analysis, and the legends of the enrolled studies were supplied.

**Correlation of OSBPL3 and TMB (tumor mutational burden) / MSI (microsatellite instability)**

Based on STATA 12.0 software (StataCorp LP, College Station, TX, USA), we used the meta function to perform a forest plot to pool the relationship between OSBPL3 and TMB / MSI in all cancer types, the P-value and hazard ratio (HR) are shown.

**Phosphorylation feature prediction**

We logged into the open-access PhosphoNET database (<http://www.phosphonet.ca/>), and obtained the predicted phosphorylation features of the S34, S197, S251, S262, S273, S410 and S437 sites by searching the protein name “OSBPL3”.

**Immune infiltration analysis**

We downloaded more than forty commonly immune-related checkpoints genes from the TCGA database in various tumors and extract the expression values of these genes and the expression of OSBPL3. *, *P*<0.05; **, *P*<0.01; ***, *P*<0.001.

# Supplementary figure legends

**Supplementary Figure 1.** **Structural characteristics of OSBPL3 in different species and the phylogenetic tree of OSBPL3.** (A) Genomic location of human OSBPL3. (B) Conserved domains of OSBPL3 protein among different species. (C) We used a constraint-based multiple alignment tool of NCBI to obtain the phylogenetic tree of OSBPL3 in different species.

**Supplementary Figure 2. Expression level of OSBPL3 in different cells and tissues in the normal physiological state.** (A) The expression statuses of the gene OSBPL3 in normal tissues. (B) We analyzed the expression of the OSBPL3 gene in different tissues using the consensus datasets of HPA, GTEx and FANTOM5 or in different blood cells using the consensus dataset of HPA, Monaco and Schmiedel (C).

**Supplementary Figure 3. Expression level of the OSBPL3 gene in different tumors and pathological stages.** (A) The expression statuses of the gene OSBPL3 in different tumor tissues in TCGA project were compared with the corresponding normal tissues of the GTEx databases. (B) Based on the CPTAC dataset, we also analyzed the protein expression level of OSBPL3 total protein between normal tissue and primary tissue in renal cell carcinoma, colon cancer, lung adenocarcinoma, uterine corpus endometrial carcinoma in tumor paired samples.

**Supplementary Figure 4. Pooled analysis on the OSBPL3 expression difference between normal and tumor tissues via the Oncomine database.** (A) Pancreatic cancer; (B) Lung cancer; (C) Colorectal cancer; (D) Liver cancer; (E) Cervical cancer.

**Supplementary Figure 5. Correlation between OSBPL3 expression and disease-free interval (DFI) prognosis of cancers in TCGA.** (A) We performed progression free interval analyses OSBPL3 gene expression by Kaplan-Meier curves in LGG and PAAD cancers. (B) We performed a meta-analysis (Forest Plot) for pooling of a series of univariate disease-free survival analyses by OSBPL3 expression in different tumors from TCGA. Hazard ratios, with 95% confidence intervals, and P value are shown in each different group.

**Supplementary Figure 6. Correlation between OSBPL3 expression and tumor mutational burden / microsatellite instability.** Spearman correlation analysis of TMB (A) /MSI (B) and OSBPL3 gene expression. The horizontal axis in the figure represents the correlation coefficient between genes and TMB/MSI, the ordinate is different tumors, the size of the dots in the figure represents the size of the correlation coefficient, and the different colors represent the significance of the p value. The bluer the color, the smaller the *P* value.

**Supplementary Figure 7. Correlation between OSBPL3 expression and immune infiltration in different tumors.** We analyzed the relationship between the expression level of OSBPL3 and the Est_ImmuneScore based on ESTIMATE algorithm in different tumors from TCGA database.

**Supplementary Figure 8. Correlation between OSBPL3 expression and stromal infiltration in different tumors.** We analyzed the relationship between the expression level of OSBPL3 and the “StromalScore” based on ESTIMATE algorithm in different tumors from TCGA database.

**Supplementary Figure 9. Correlation between OSBPL3 expression and comprehensive score of immune cells and stromal cells in different tumors.** We analyzed the relationship between the expression level of OSBPL3 and the “ESTIMATEScore” based on ESTIMATE algorithm in different tumors from TCGA database.

**Supplementary Figure 10. GO analysis of OSBPL3-related genes in tumors.** (A-C) The cnetplot for the GO analysis**---**biological process (BP) / molecular function (MF)/ cellular component (CC) is also shown.

**Supplementary Table**

**Table 1. Abbreviation of tumors.**

| Abbreviation | Tumors |
| --- | --- |
| ACC | Adrenocortical carcinoma |
| BLCA | Bladder Urothelial Carcinoma |
| BRCA | Breast invasive carcinoma |
| CESC | Cervical squamous cell carcinoma and endocervical adenocarcinoma |
| CHOL | Cholangiocarcinoma |
| COAD | Colon adenocarcinoma |
| READ | Rectum adenocarcinoma Esophageal carcinoma |
| DLBC | Lymphoid Neoplasm Diffuse Large B-cell Lymphoma |
| ESCA | Esophageal carcinoma |
| GBM | Glioblastoma multiforme |
| HNSC | Head and Neck squamous cell carcinoma |
| KICH | Kidney Chromophobe |
| KIRC | Kidney renal clear cell carcinoma |
| KIRP | Kidney renal papillary cell carcinoma |
| LAML | Acute Myeloid Leukemia |
| LGG | Brain Lower Grade Glioma |
| LIHC | Liver hepatocellular carcinoma |
| LUAD | Lung adenocarcinoma |
| LUSC | Lung squamous cell carcinoma |
| MESO | Mesothelioma |
| OV | Ovarian serous cystadenocarcinoma |
| PAAD | Pancreatic adenocarcinoma |
| PCPG | Pheochromocytoma and Paraganglioma |
| PRAD | Prostate adenocarcinoma |
| READ | Rectum adenocarcinoma |
| SARC | Sarcoma |
| SKCM | Skin Cutaneous Melanoma |
| STAD | Stomach adenocarcinoma |
| STES | Stomach and Esophageal carcinoma |
| TGCT | Testicular Germ Cell Tumors |
| THCA | Thyroid carcinoma |
| THYM | Thymoma |
| UCEC | Uterine Corpus Endometrial Carcinoma |
| UCS | Uterine Carcinosarcoma |
| UVM | Uveal Melanoma |
